# A systematic scoping review of the use of surfactant replacement therapy for respiratory distress syndrome in preterm neonates in low- and middle-income countries

**DOI:** 10.3389/fped.2025.1685625

**Published:** 2025-11-07

**Authors:** Caris A. Price, Lloyd Tooke, Heather J. Zar, Brenda M. Morrow

**Affiliations:** 1 Department of Paediatrics and Child Health, University of Cape Town, Cape Town, South Africa; 2 SA-MRC Unit on Child and Adolescent Health, University of Cape Town, Cape Town, South Africa

**Keywords:** surfactant, preterm, neonate, respiratory distress syndrome, resource-limited settings, low- and middle-income countries

## Abstract

**Introduction:**

The availability and use of surfactant replacement therapy (SRT) for respiratory distress syndrome (RDS) in low- and middle-income countries (LMICs) is variable with unclear impact on infant outcomes. This review evaluates the published evidence on SRT in the management of preterm neonates with RDS in LMICs, with a focus on SRT availability, administration, timing, type, and cost.

**Methods:**

A systematic scoping review of seven databases was conducted, following the Preferred Items for Systematic Reviews and Meta-Analysis guidelines extension for Scoping Reviews. English language systematic reviews and observational and experimental studies, published between January 2010 and July 2023, were eligible for this review. Case reports, small case series, and qualitative studies were excluded. Titles and abstracts were screened by one reviewer and full text by two independent researchers. Sufficiently homogeneous randomized controlled trials (RCTs) were synthesized using random-effects meta-analyses, while other results were synthesized narratively. Primary outcomes for meta-analyses were (1) need for invasive mechanical ventilation (IMV), (2) development of bronchopulmonary dysplasia (BPD), and (3) in-hospital mortality.

**Results:**

After screening 483 titles/abstracts and 266 full texts, 113 articles were included in the final review (52 RCTs, 50 observational studies, and 11 systematic reviews). Studies reported both INtubation-SURfactant-Extubation (INSURE) and Less/Minimal Invasive Surfactant Administration/Treatment (LISA/MIST) methods of SRT, with different threshold criteria for implementation. There was moderate certainty evidence that using LISA/MIST reduced the need for IMV [risk difference (RD): 0.10 (95% confidence interval, CI: 0.04–0.17); *p* = 0.001] compared with INSURE, with a borderline effect on BPD [RD: 0.04 (95% CI: 0.00–0.08); *p* = 0.05] and no significant effect on mortality [RD: 0.01 (95% CI: −0.02 to 0.04; *p* = 0.5)]. There was low certainty evidence that poractant alfa (200 mg/kg) was associated with a reduced need for mechanical ventilation compared with beractant (100 mg/kg) (RD 0.10 (95% CI: 0.02–0.18); *p* = 0.01), with a similar reduction in mortality [RD: 0.07 (95% CI: 0.01–0.13); *p* = 0.02]. No cost-effectiveness studies were identified.

**Conclusion:**

LISA/MIST should be used in preference to INSURE. Poractant alfa (200 mg/kg) is conditionally recommended in preference to beractant (100 mg/kg). Regionally relevant cost-effectiveness studies are needed.

## Introduction

A leading cause of neonatal mortality (NNM), preterm birth and associated complications, result in approximately 1 million infant deaths annually, 99% of which occur in low- and middle-income countries (LMICs). NNM, accounting for almost 50% of deaths in children under 5 years (1), accounts for an increasing proportion of deaths, as other causes have greatly reduced in the past few decades. While the contribution of NNM to childhood deaths increases, more neonates are born [and in high-income countries (HICs), surviving] at an even younger gestational age (GA) (2). Achieving the target of fewer than 12 neonatal deaths per 1,000 births, as stipulated in the Sustainable Development Goals (SDGs) of 2030 (3), will require a strengthening of antenatal and perinatal care.

Approximately 45% of prematurity-related deaths in LMICs are attributable to respiratory distress syndrome (RDS) (4) caused by lung immaturity and surfactant deficiency (5). Respiratory interventions such as continuous positive airway pressure (CPAP) and surfactant replacement therapy (SRT) have improved the outcomes of preterm infants with RDS and are part of standard care in HICs (6, 7). However, in 2020, only 33% of African countries had access to SRT and 63% to CPAP (6). Even in settings where these interventions are available, costs may be prohibitive (6, 8–18). Furthermore, most of the evidence supporting their use is from HICs (14, 16, 19, 20), with a lack of data from Africa and other LMICs. Inaccurate gestational age assessments, inadequate antenatal care and underutilization of antenatal steroids, urban–rural and public–private resource discordance, and wide variation in the number of live births occurring outside health facilities further complicate a standardized SRT approach in resource-limited LMIC settings.

In 2016, Sankar et al. (21) published a seminal systematic review on the efficacy, safety, feasibility, and cost-effectiveness of SRT in low-resource settings. The review confirmed a significant reduction in both mortality and risk of air leaks in preterm neonates who received SRT. Suggested areas for future research included defining ideal timing for rescue SRT, its cost- effectiveness, exploration of less invasive modalities of SRT, and the effect of SRT on bronchopulmonary dysplasia (BPD) in resource-restricted settings (RRS) (21).

A growing body of research in LMICs around the threshold criteria, ideal timing, surfactant type and dose, method of administration, and respiratory support pre- and post-SRT has emerged in the last decade. Studies in HICs have demonstrated that Less Invasive Surfactant Administration (LISA) reduces the composite outcome of death or BPD compared with the INtubation-SURfactant-Extubation (INSURE) method (22).

This systematic scoping review aimed to provide an update on the availability and practices of SRT and their effect on infant outcomes in LMICs, and to make recommendations for use, to inform policy and identify priority research areas.

## Methods

This study was a systematic scoping review (23) of the published literature on the use of SRT in the treatment of RDS in preterm neonates in LMICs. The objectives were (1) to explore the availability and use of SRT in LMICs and describe the range of current practice and (2) to describe the impact of SRT on infant outcomes, including the need for invasive mechanical ventilation (IMV), on BPD prevalence, and on mortality.

### Protocol and registration

A scoping review protocol was created *a priori*. This paper adheres to Preferred Items for Systematic Reviews and Meta-Analysis guidelines extension for Scoping Reviews (PRISMA-ScR) (24), and as per these guidelines, registration as a systematic review was not required.

### Ethics

An ethical waiver was granted on the grounds that “As the systematic review involves published literature available through publicly accessible electronic databases, research ethics review and approval is not required” (HREC REF 301/2021).

### Eligibility criteria

Published systematic reviews and observational and experimental studies that focused on SRT, with or without ancillary therapies, in preterm neonates with or at-risk of RDS, conducted in LMICs as defined by World Bank Income (WBI) categories (25), were considered for inclusion. Specifically, studies describing the availability, use (criteria for use, method, and timing of administration), complications, in-hospital outcomes, and mortality associated with SRT in these settings were considered for inclusion. Case reports, case series, and qualitative studies were excluded. Unpublished data or research in progress were not included.

### Information sources

Primary systematic searches were conducted in EBSCOhost (Africawide and CINAHL), Web of Science, Scopus, Scielo, and Cochrane, using comprehensive search strategies with controlled vocabulary and Boolean operators, while Google Scholar was used supplementarily to capture literature not indexed in traditional databases and identify additional sources through citation searching, ensuring breadth of coverage appropriate for a scoping review's exploratory aims. Peer-reviewed literature published in English between January 2010 and June 2025 and meeting eligibility criteria was considered for inclusion. The final and most recent search was executed on 9 October 2025.

### Search strategy

Search strategies were customized for each database's indexing system while maintaining core medical subject headings (MeSH) terms. The filters used included date and language limits as specified above. The full search strategy for PubMed was

#1 “Pulmonary Surfactants"[Mesh] OR “Surface-Active Agents"[Mesh]

#2 surfactant*[tiab] OR “surface active agent*"[tiab] OR “lung surfactant*"[tiab] OR exogenous surfactant*[tiab] OR beractant[tiab] OR survanta[tiab] OR poractant[tiab] OR curosurf[tiab] OR calfactant[tiab] OR infasurf[tiab] OR bovactant[tiab] OR lucinactant[tiab]

#3 #1 OR #2

#4 “Respiratory Distress Syndrome, Newborn"[Mesh]

#5 “respiratory distress syndrome"[tiab] OR RDS[tiab] OR “hyaline membrane disease"[tiab] OR HMD[tiab] OR “neonatal respiratory distress"[tiab]

#6 #4 OR #5

#7 “Infant, Premature"[Mesh] OR “Infant, Extremely Premature"[Mesh] OR “Infant, Very Low Birth Weight"[Mesh] OR “Infant, Extremely Low Birth Weight"[Mesh] OR “Infant, Low Birth Weight"[Mesh]

#8 preterm*[tiab] OR premature*[tiab] OR “low birth weight"[tiab] OR LBW[tiab] OR VLBW[tiab] OR ELBW[tiab] OR “very low birth weight"[tiab] OR “extremely low birth weight"[tiab]

#9 #7 OR #8

#10 “Developing Countries"[Mesh] OR “Africa"[Mesh] OR “Asia"[Mesh] OR “South America"[Mesh] OR “Central America"[Mesh] OR “Caribbean Region"[Mesh]

#11 LMIC*[tiab] OR “low income countr*"[tiab] OR “middle income countr*"[tiab] OR “low and middle income"[tiab] OR “developing countr*"[tiab] OR “developing nation*"[tiab] OR “developing world"[tiab] OR “less developed countr*"[tiab] OR “under developed countr*"[tiab] OR “underdeveloped countr*"[tiab] OR “third world"[tiab] OR “low resource"[tiab] OR “limited resource"[tiab] OR “resource limited"[tiab] OR “resource poor"[tiab]

#12 [LIST OF INDIVIDUAL LMIC COUNTRIES]

#13 #10 OR #11 OR #12

#14 #3 AND #6 AND #9 AND #13

Filters: Humans

### Selection of sources of evidence

**Figure 1:**
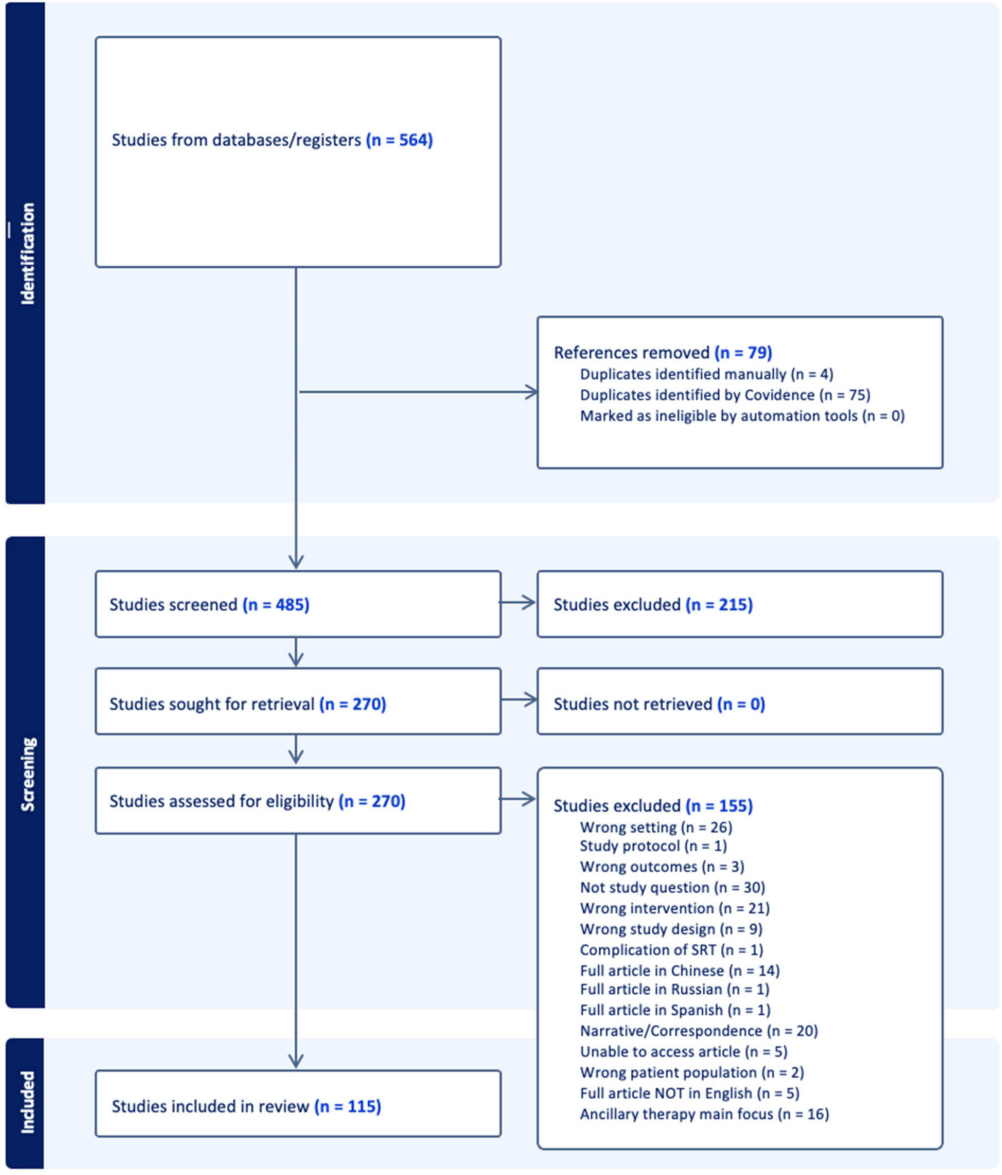
PRISMA diagram of study flow after initial title screening.

Following the search, identified citations were uploaded into both *EndNote* (26) and Covidence (https://www.covidence.org/), and duplicates were removed. One reviewer screened titles and abstracts against the inclusion criteria to identify potentially relevant studies. Once retrieved, full texts were reviewed for inclusion by two independent researchers. The online software program, Covidence systematic review software (Veritas Health Innovation, Melbourne, Australia, 2023), was used for the article screening and selection process. Any disagreements arising between reviewers were resolved through discussion and/or with an additional reviewer/s in the case of disagreement. The results of the search and study inclusion process, as well as reasons for exclusion of sources of evidence at full text review, were recorded and reported in PRISMA-ScR flow diagram (Figure 1) (24).

### Data charting process

Data from included studies were extracted in a standardized manner, using a self-developed data extraction form and charted in Microsoft Excel (Microsoft Corporation, 2018) for analysis.

### Data items

The extracted data included the following: (a) country, (b) World Health Organization (WHO) region, (c) WBI group, (d) setting (public/private, tier of healthcare, etc.), (e) study design and grouping, (f) time frame, (g) ethics approval, (h) baseline characteristics (overall sample, intervention group, control group), (i) inclusion criteria, (j) exclusion criteria, (k) surfactant details [type, dosage, indications, timing, method of administration, use of premedication, rank of healthcare provider performing SRT, number of attempts of SRT, mean time dose(s) given, definition of SRT failure, implementation strategy, barriers and facilitators for use], (l) characteristics of ancillary therapies [including non-invasive ventilation (NIV), IMV, antenatal steroid use, methylxanthine use, postnatal steroid use, oxygen therapy, settings used, etc.], and (m) outcomes, including mortality and other adverse events.

### Critical appraisal of individual sources of evidence

In the case of studies being amenable to pooling results with meta-analyses, a combination of the “Grading of Recommendations, Assessment, Development, and Evaluations” (GRADE) criteria (27) and Joanna Briggs Institute (JBI) critical appraisal tool checklists (for observational studies) (23) were applied to inform the certainty of results (see the summary of findings’ tables). The Robvis tool (28) was used to generate critical appraisal infographics for pooled randomized controlled trials (RCTs).

### Synthesis of results

While the primary purpose of this study was a scoping review to map the literature on the topic, secondary meta-analyses of homogeneous RCTs were conducted after assessing for clinical and methodological homogeneity. Clinical homogeneity was evaluated by comparing study populations (gestational age, birth weight), interventions (surfactant type, dose, delivery method), comparators, and outcome definitions. Methodological homogeneity was assessed by examining study design quality, risk of bias, and outcome measurement approaches. Sufficiently homogeneous RCTs, considering interventions, outcomes, and study sample, were synthesized using random-effects meta-analyses, while other results were synthesized narratively. Primary outcomes for meta-analyses were (1) progression to IMV, (2) development of BPD, and (3) in-hospital mortality.

Statistical heterogeneity was subsequently assessed using the *I*^2^ statistic *post hoc*, with *I*^2^ > 50% considered substantial heterogeneity. When substantial statistical heterogeneity was detected despite clinical homogeneity, we planned to explore potential sources through sensitivity or subgroup analyses. Certainty of evidence was assessed using GRADE methodology, with heterogeneity contributing to downgrading when *I*^2^ > 50%. IBM SPSS Statistics (version 28.0.1.1) was used for meta-analysis, and a *p*-value of <0.05 was considered statistically significant.

## Results

### Selection of sources of evidence

A total of 975,639 articles were identified on the initial search, the titles of which were screened by the first author. Following title screening, 564 citations were exported to Covidence. After removing duplicates, 485 titles and abstracts were screened with a resultant 265 full texts assessed for eligibility. After excluding 150 studies, 115 articles were included in the final review (Figure 1). The breakup for these articles was 53 RCTs, 11 systematic reviews, and 51 observational studies (Supplementary Appendix 1, Supplementary Tables S1–S3).

### Characteristics of the sources of evidence

**Figure 2:**
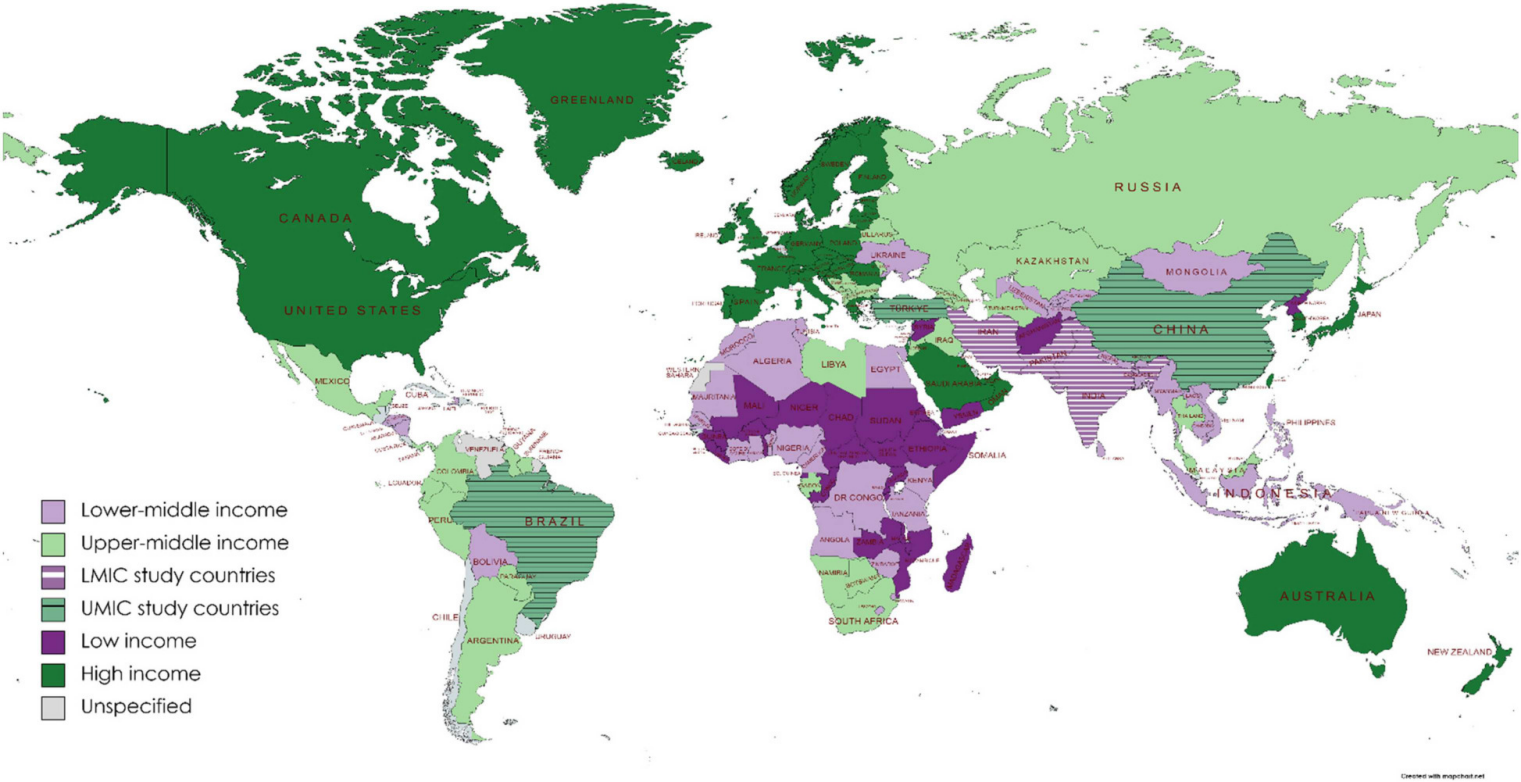
Distribution of publications by World Bank Income Groups.

**Figure 3:**
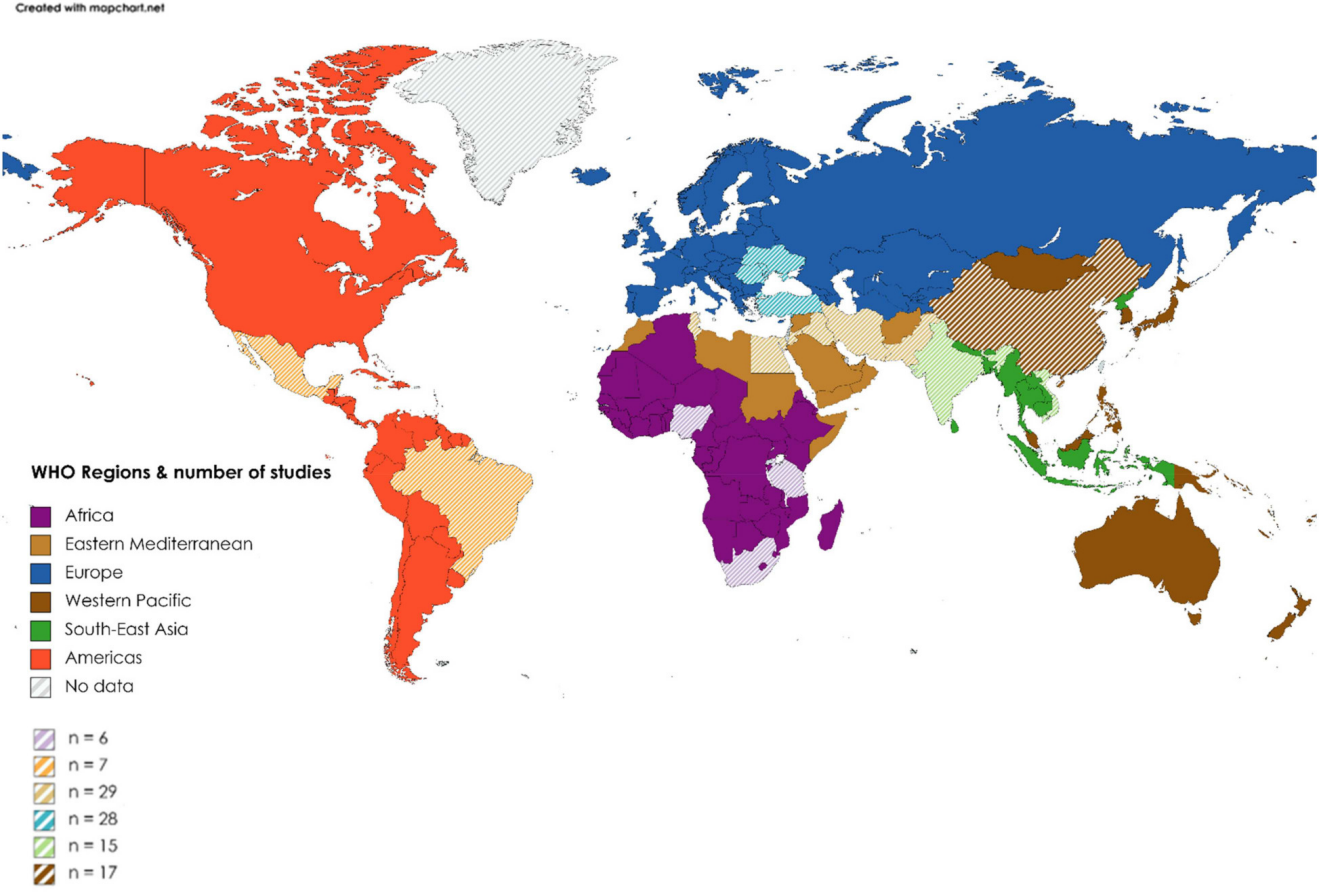
Distribution of publications by WHO region. Color, WHO region; Striped areas, countries from region that contributed to publication count (see map key for the total number of publications per WHO region).

Excluding systematic reviews (*n* = 11), most (74%) articles were published between 2014 and 2017 (33%) and 2018 and 2021 (41%), with a trend to an increasing number of publications over time. In terms of WBI group classification (25) (Figure 2), 44% (*n* = 45) of studies originated from low- to middle-income countries and the remainder from high-to middle-income countries. Most publications were from the WHO regions of Eastern Mediterranean (*n* = 29; 28.4) or Europe (*n* = 28; 27.5%), with the African region publishing only 6 (5.9%) studies (Figure 3). Study methodology was almost equally split between RCTs (46%) and observational (44%) designs, with only one each of “cost-effectiveness” and “diagnostic accuracy” studies. Studies were conducted in 19 countries, most frequently Turkey (24.5%), followed by Iran (22.5%), with many countries producing only one included study.

A total sample of 6,524 infants was derived from all included RCTs. Sample sizes varied widely per study, with most RCTs utilizing samples of between 40 and 99 participants and most observational studies including ≥200 infants. Studies were only included if at least a proportion of the population were likely to be premature neonates; however, the range of gestational ages was highly heterogeneous. Many studies also used infant birth weight as an inclusion criterion, with most using 500 g as a lower limit. The male and female genders were mostly equally represented in the samples. Most studies were conducted in level III neonatal intensive care units (NICUs) and typically under the auspices of specialist pediatricians or neonatologists.

The most frequent research question pertained to the method of administration of SRT (24.5%), with most comparing less invasive methods vs. endotracheal administration. Other questions covered the topics of initial respiratory support strategies prior to and/or mitigating the need for SRT (11.8%) and post-SRT respiratory support strategies (4.9%), timing of SRT (8.8%), surfactant type (15.7%), and SRT augmentation (3.9%), while one study addressed the topic of peri-SRT sedation. With regard the outcomes of interest, the need for IMV was the most commonly reported outcome (48%), followed by mortality (26.4%) and BPD incidence (17.6%) (Table 1).

**Table 1:** Study designs and primary outcomes.

Research question	RCTs, *n* (%) *n* = 52	Observational, *n* (%) *n* = 50	Total, *n* (%) *n* = 102 (*γ*)
Initial respiratory support strategy	6 (11.5)	6 (12)	12 (11.8)
Timing of SRT	4 (7.7)	5 (10)	9 (8.8)
Surfactant type	14 (26.9)	2 (4)	16 (15.7)
Method of SRT administration	18 (34.6)	7 (14)	25 (24.5)
Sedation/premed	1 (3.1)	0	1 (1)
SRT augmentation	4 (7.7)	0	4 (3.9)
Post-SRT respiratory support strategy	4 (7.7)	1 (2)	5 (4.9)
Impact of SRT			
Primary outcome^a^	RCTs	Observational	Total *n* (%) *n* = 102
Need for IMV	30 (57.6)	19 (38)	49 (48)
BPD incidence	3 (5.7)	15 (30)	18 (17.6)
Mortality	1 (1.9)	26 (52)	27 (26.4)

SRT, surfactant replacement therapy; IMV, invasive mechanical ventilation; BPD, bronchopulmonary dysplasia; γ, excluding systematic reviews (113 − 11 = 102).

a Some studies included more than one primary outcome.

### Critical appraisal within sources of evidence

Studies that were pooled for meta-analysis displayed generally low levels of bias (see Supplementary Appendix 2, Supplementary Figures S1–S3). Limitations may include selection bias given that all non-English publications were excluded, for pragmatic reasons. In addition, despite certain groups of studies being amenable to pooling for meta-analysis, heterogeneity was present and rendered evidence of moderate to low certainty.

## Synthesis of findings

### Availability and use of SRT in LMICs

The most well-represented countries were Turkey and Iran. No randomized controlled studies were identified from any African country. Several papers reported that surfactant, while available, is often not readily accessible because of its prohibitive cost (6–18). The coverage of surfactant in African countries is estimated to be <40% (29), despite its inclusion in the WHO Essential Drug List. In addition, surfactant is generally used only in centers that are also able to offer mechanical ventilation (13), and the availability (14, 15, 17) thereof is an additional challenge. Even supplemental oxygen and x-ray facility availability (15, 16) were far from ubiquitous. A lack of national health insurance schemes or severe limitations therein mean that in some countries, surfactant and any ancillary therapy are given only where *parents* can afford it, out of pocket (9, 13). In certain settings, the cost of SRT exceeds the average per capita Gross National Product (GNP) of the country (9), rendering “cost rather than care” (9) the driver of SRT.

### Threshold criteria/indications for SRT

**Figure 4:**
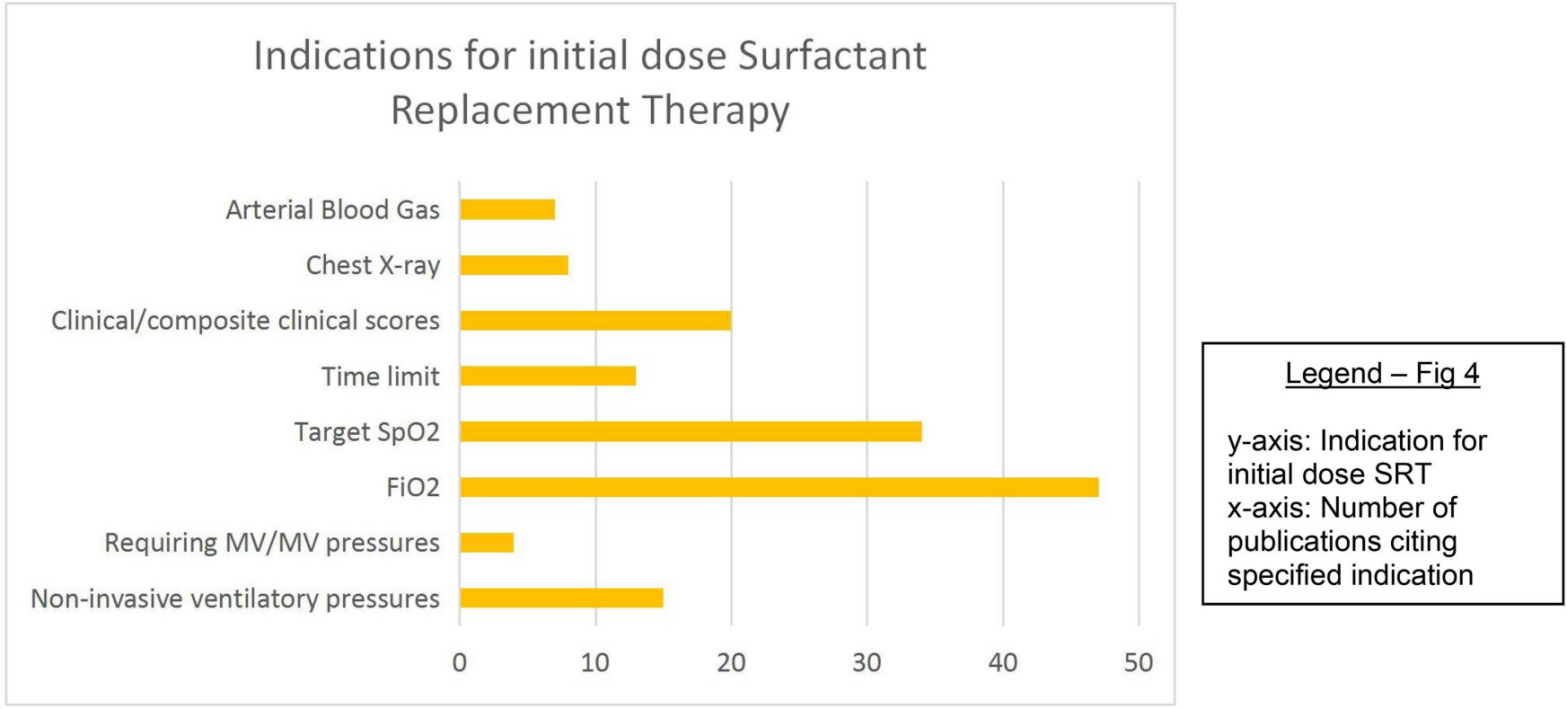
Indications for initial dose of SRT.

Fifty-six percent of studies described the threshold at which the *first* dose of SRT was administered. These include, in order of descending frequency: fraction of inspired oxygen (FiO_2_), target oxygen saturation (SpO_2_), clinical features or composite clinical score [e.g., Silverman Anderson score (30), Acute Care of At-Risk Newborns (ACoRN) (31)], non-invasive ventilatory pressure cut-off values, time limits, radiographic findings, arterial blood gas analysis, and the requirement for IMV/IMV pressure cut-off values. Most studies used a combination of these factors, especially FiO_2_, target SpO_2_, and time limits (Figure 4). Vardar et al. (32) developed a lung ultrasound (LUS) severity score [reference standard called chest radiograph (CXR)], which predicted the need for administering initial and subsequent surfactant doses with promising specificity and sensitivity.

A FiO_2_ threshold of requiring ≥0.4 to meet target saturation was most frequently employed as the indication to provide SRT, with the commonest target saturation range being 90%–95%. Eight studies (22, 33–39) used a target saturation range with a lower limit of 85%, and four studies (40–43) used a FiO_2_ threshold of ≥0.50.

### Type and dose of surfactant

Sixteen studies (14 RCTs) compared different types of surfactants for several outcomes (8, 14, 33, 41, 44–55). Only one retrospective study (14) compared natural with synthetic surfactant; the remainder compared naturally derived surfactants. Sixty percent of these studies compared beractant (Survanta, AbbVie Laboratories, Chicago, IL, USA) and poractant alfa (Curosurf, Chiesi Farmaceutici, Parma, Italy) (eight RCTs and one observational study).

Excluding cost-effectiveness and diagnostic studies, guidelines, systematic reviews, and studies directly comparing surfactant types not used as standard practice, 73.5% of original studies specified either the type or dose, or both, of the initial bolus of surfactant routinely used in their settings or study. Of those where the type was known, porcine surfactant was used in 60%, bovine in 34.5%, and either bovine or porcine (depending on availability) in 5.5%. Supplementary Tables S4 and S5 display the various types and trade names of surfactants used in the included studies. The most frequent type/dose combination used was poractant alfa (200 mg/kg), followed by beractant (100 mg/kg), according to the manufacturer's recommended dosages.

Kandraju et al. (56) mentioned the lack of availability of Curosurf in the Indian market for a few months during the authors' study period, while an additional four studies (6, 9, 42, 57) indicated that the type of surfactant used was entirely dependent on availability.

### Method of administration

Seventeen RCTs (22, 40, 58–73) and six observational studies (37, 66, 74–77) compared various methods of surfactant administration (Supplementary Appendix 1, Supplementary Tables S1, S2) generally, and “less invasive” methods [predominantly LISA but also use of a laryngeal mask airway (LMA) (40)/via thin intratracheal catheter (CATH) (59)/Minimally Invasive Surfactant Administration (MISA) (67)/Minimal Invasive Surfactant Treatment (MIST) (63, 70, 72)/TakeCare (61)] involving either a feeding, vascular, or other catheter as an intratracheal conduit for SRT were compared with intratracheal administration via the endotracheal tube (ETT) for determining several outcomes, including those pertinent to this review.

Excluding those studies that compared two methods of administration, LISA alone was used in 11.7% of studies, INSURE in 51.9,; either/or both these techniques in 3.9%, and the method was not specified in 32.4%.

Reported strengths of the LISA technique include the following: it is relatively easy to learn (22) and the use of an infant feeding tube as a conduit is cost-effective and immediately available (68); while other studies report concerns regarding procedural sedation (or lack thereof) (63, 70, 78) and staff reluctance to transition to this method (76).

As shown in Supplementary Tables S1 and S2, heterogeneity existed between studies in terms of the type and size of the intratracheal conduit used, its depth of insertion, the type of NIV maintained during the less invasive procedure, and the exact methodology used to deliver SRT. The control procedures also differed.

### Timing of administration

Ten studies [four RCTs (56, 79–81) and six observational (75, 82–86)] investigated the role of timing of SRT on outcomes, including the need for IMV, mortality, and persistence of patent ductus arteriosus (PDA). SRT timing ranged from “with the first breath” to up to 72 h after birth. No two studies tested the same window of time. Included studies broadly divided the administration into “prophylactic” use (given soon after birth, usually based on GA) or “rescue” use (given once certain clinical parameters met). Most units appear to be using clinical indications, rendering time a secondary consideration. The extreme heterogeneity of the data precluded meta-analysis. Five of the studies (56, 81–83, 86) demonstrated statistically significant findings, with “early” surfactant having more favorable outcomes than “late” SRT.

### SRT augmentation

Four RCTs (87–90) described the application of SRT augmentation with 75% of these originating from Iran. Two (88, 90) described intratracheal therapy in addition to intratracheal surfactant—one (88) reported the instillation of budesonide 0.25 mg/kg, while the other (90), salbutamol 0.2 mg/kg. A third study (89) described nebulization of the infant with salbutamol 0.15 mg/kg by a micropump nebulizer 10 min prior to SRT. The fourth study (87) described the addition of salbutamol 0.2 mg/kg, although it was not clear by what route this was administered. In both studies comparing salbutamol (89, 90) with a control, the need for IMV was significantly lower in the intervention group. The incidence of BPD in the SRT + budesonide group was significantly lower than that in the control group (88). However, the samples were small in size, BPD definitions were not standardized (limiting generalizability), and the quality of evidence was poor. Neurodevelopmental (and other long-term) follow-up was lacking, and further research in RRS is required.

### Respiratory support strategies—pre- and post-SRT

Fourteen studies—10 RCTs (43, 91–99) and 4 observational studies (34, 57, 100, 101)—examined the mode of respiratory support either/both prior to and following SRT. Most compared the use of NIV *other than CPAP* [duo positive airway pressure (DUOPAP), bilevel positive airway pressure (BiPAP), and non-invasive intermittent positive pressure ventilation (nIPPV)] or non-invasive high frequency oscillatory ventilation (nHFOV) vs. CPAP, with a small number comparing invasive with non-invasive ventilation or high flow nasal cannula (HFNC) with other respiratory support.

### Premedication practices

Excluding systematic reviews, eight studies (19, 40, 50, 59, 71, 88, 102, 103) reported the use of premedication or analgesia during SRT: 22.2% with LISA, 22.2% with INSURE, and 55.6% with both/either method of delivery. Premedication or analgesia was not reported in 75% of included studies. Fourteen studies (34, 37, 56, 61, 64, 65, 67–69, 74, 75, 77, 79, 80) reported the use of no premedication or analgesia, and four studies (68, 69, 72, 102) reported the use of non-pharmacological measures such as nesting and swaddling during SRT delivery. A variety of drugs were employed by those using pharmacological measures, including fentanyl (50, 88, 102), atropine (59), morphine (71), combinations of remifentanil (40) and midazolam (40), or atropine and ketamine (103), phenobarbitone (19), and in the case of the LMA vs. LISA, lidocaine gel (40).

## Outcomes

A lack of standardization with regard to terminology, procedures, and outcome measures rendered much of the data inhomogeneous and meaningfully incomparable. However, sufficiently homogeneous studies were pooled in meta-analyses for the primary outcomes of this review. The only factor found to impact the outcome of mortality, with moderate certainty of evidence, was that of poractant alfa over beractant.

### Type of surfactant

**Figure 5:**
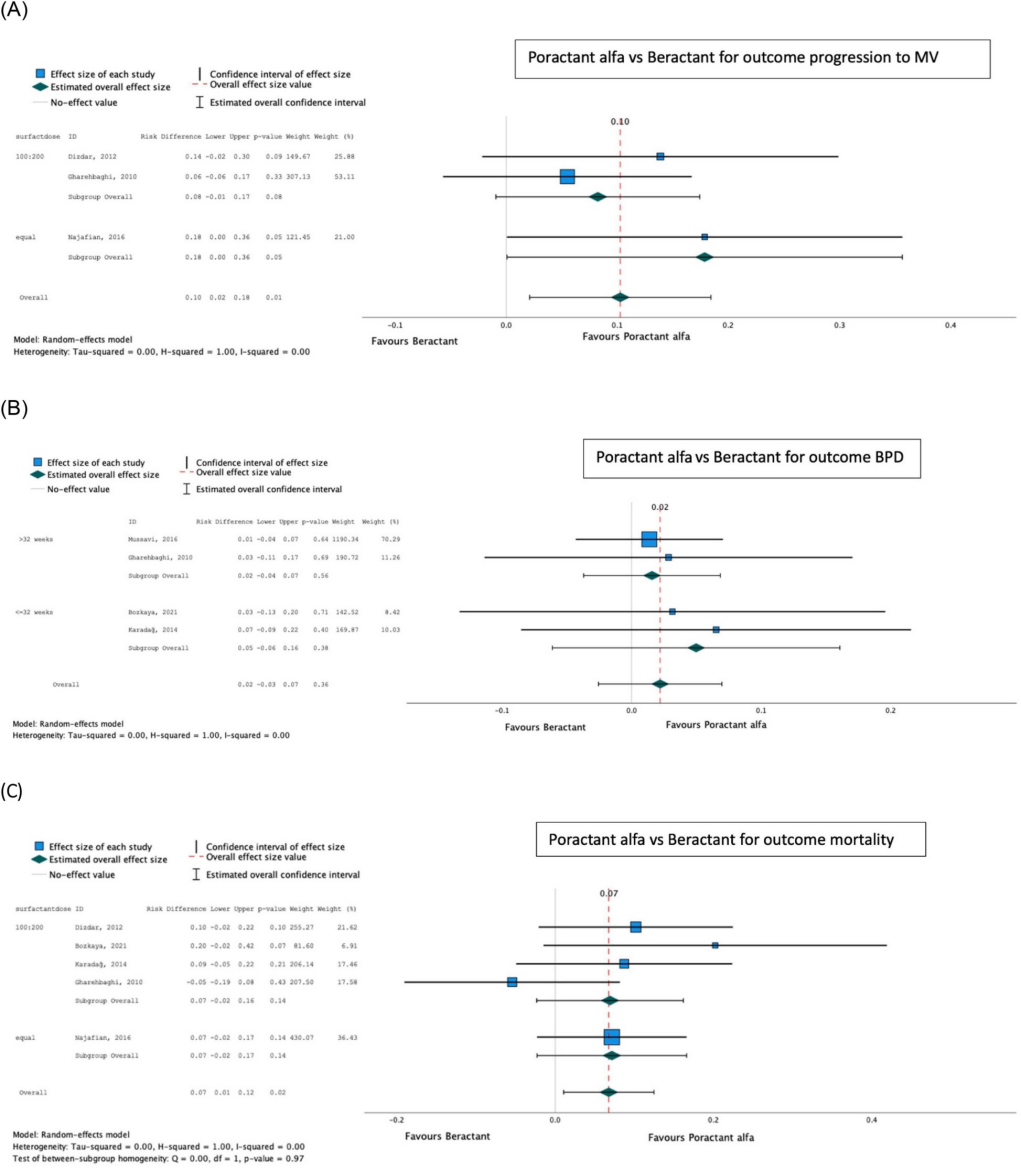
Effect of poractant alfa vs. beractant on progression to invasive mechanical ventilation (**A**), bronchopulmonary dysplasia (**B**), and mortality (**C**).

Poractant alfa was found to be superior to beractant for mortality [pooled risk difference (95% confidence interval, CI) 0.07 (0.01–0.12); *p* = 0.02] and progression to IMV (pooled risk difference 0.10 (0.02–0.28); *p* = 0.01), with no difference in the proportion developing BPD [pooled risk difference 0.02 (−0.03 to 0.07); *p* = 0.36] (Table 2; Figure 5). There was no significant difference between dosing schedules or gestational age groups on *post hoc* sensitivity analysis.

**Table 2:** Poractant alfa vs. beractant and outcomes.

Outcomes	Absolute effects		Relative effect			
	Intervention poractant alfa	Control beractant	Effect size risk difference (95% CI)	Number of participants (studies)	*P*-value	Certainty of evidence (GRADE)
Progression to invasive MV^c^	209.18/1,000 population	333.33/1,000 population	0.10 (0.02 to −0.18)	388 (3)	0.01	Low^a^
Mortality	154.12/1,000 population	215.33/1,000 population	0.07 (0.01 to −0.13)	553 (5)	0.02	Moderate
BPD^b^	148.14/1,000 population	172.09/1,000 population	0.02 (−0.03 to −0.07)	431 (4)	0.36	Low^a^

a Downgraded twice for small number of included studies with very wide CIs.

b All doses 200 mg/kg poractant alfa vs. 100 mg/kg beractant.

c No samples specifically ≤32 weeks. A sensitivity analysis for differential dosing strategies showed that the subgroups were homogeneous (*p* > 0.1) with no significant differences in outcomes.

### Method of administration

**Figure 6:**
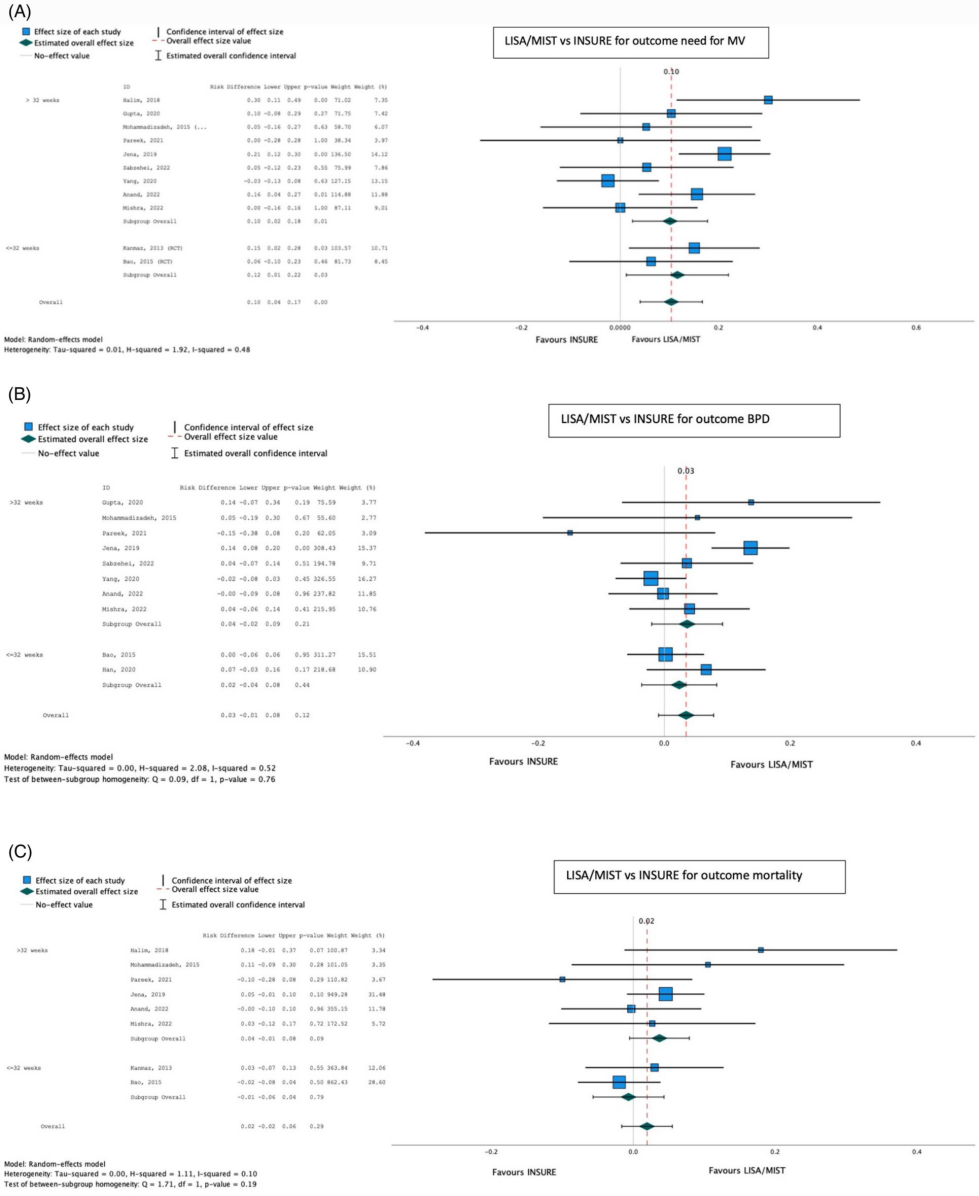
Effect of LISA/MIST vs. INSURE on progression to invasive mechanical ventilation (**A**), bronchopulmonary dysplasia (**B**), and mortality (**C**).

LISA/MIST was favored over INSURE for the outcome progression to IMV [pooled risk difference (95% CI) 0.10 (0.04–0.17); *p* < 0.001], while no significant difference was found between the techniques for the development of BPD [pooled risk difference 0.03 (−0.01 to 0.08); *p* = 0.12] or mortality [pooled risk difference 0.02 (−0.02 to 0.06); *p* = 0.29] (Table 3; Figure 6).

**Table 3:** LISA vs. INSURE and outcomes.

Outcomes	Absolute risk		Relative effect			
	Intervention (LISA/MIST)	Control (INSURE)	Effect size risk difference (95% CI)	Number of participants (studies)	*P*-value	Certainty of evidence (GRADE)
Progression to invasive MV	225.4/1,000 population	350.6/1,000 population	0.10 (0.04 to 0.17)	1,385 (11)	0.001	Moderate
Mortality	109.2/1,000 population	137.8/1,000 population	0.01 (−0.02 to 0.04)	1,425 (10)^a^	0.49	Moderate
BPD	105.0/1,000 population	170.3/1,000 population	0.04 (0.00 to 0.08)	1,593 (11)	0.05	Low^c^
Composite BPD/mortality^d^	201.6/1,000 population	317.8/1,000 population	0.12 (0.01 to 0.22)	258 (2)	0.03	Very low certainty^b^

a One study excluded from the final meta-analysis model owing to substantial variance.

b Downgraded thrice owing to a paucity of studies (*n* = 2), a small sample size, and a very wide CI.

c Downgraded owing to high heterogeneity (*I*^2^ > 0.5).

d All participants 32 weeks gestational age or younger.

**Figure 7:**
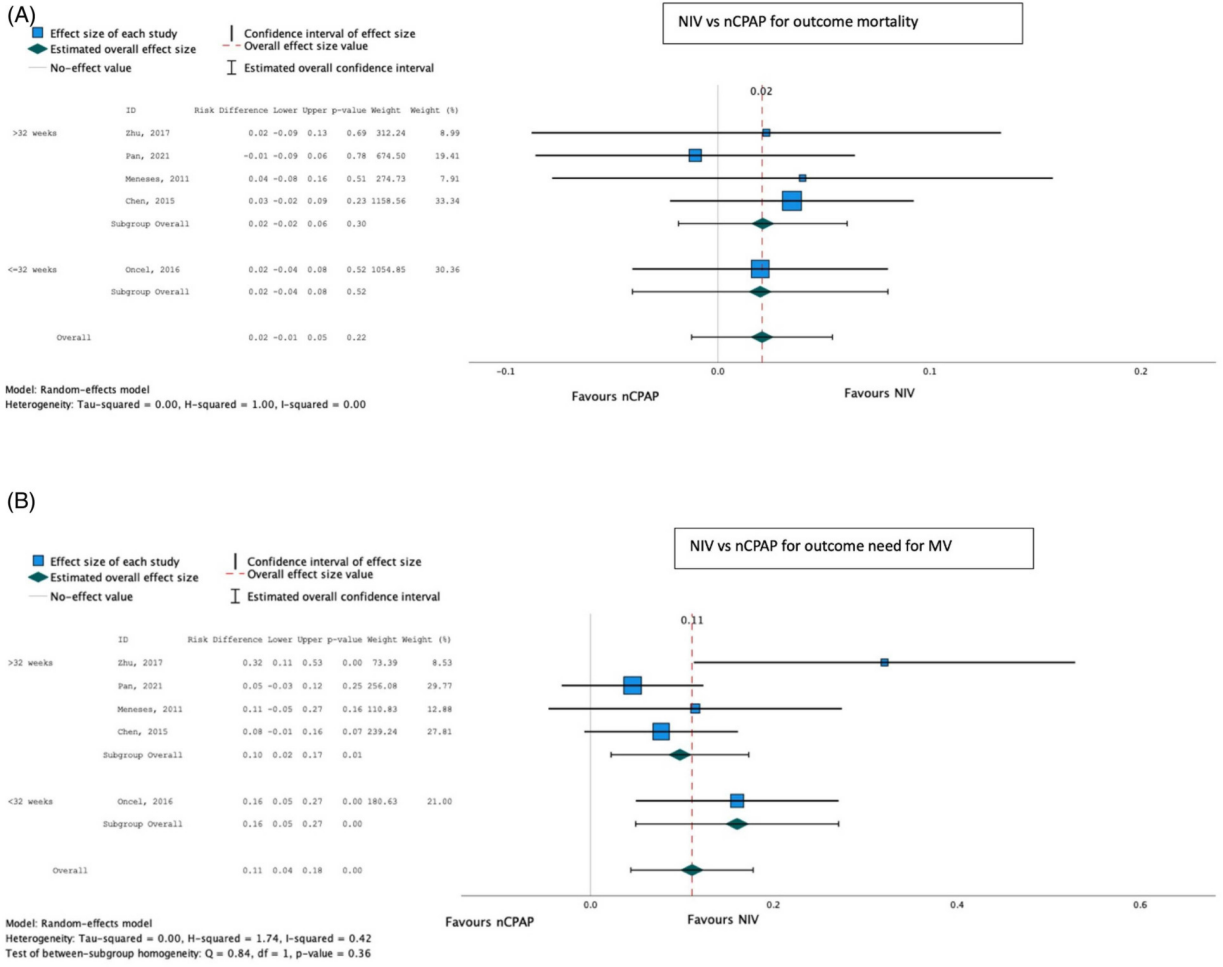
Effect of NIV other than CPAP vs. CPAP on mortality (**A**) and need for invasive mechanical ventilation (**B**).

On meta-analysis, the other methods of NIV (BiPAP, nIPPV, and nHFOV) (both pre- and post-SRT), were associated with a reduced need for IMV compared with CPAP [pooled risk difference 0.11 (0.04–0.28); *p* < 0.001], with no significant effect on mortality [pooled risk difference 0.02 (−0.01 to 0.05); *p* = 0.22] (Table 4; Figure 7).

**Table 4:** NIV vs. CPAP.

Outcomes	Absolute effects		Relative effect			
	Intervention NIV	Control nCPAP	Effect size risk difference (95% CI)	Number of participants (studies)	*P*-value	Certainty of evidence (GRADE)
Progression to invasive MV	155.87/1,000 population	266.26/1,000 population	0.11 (0.44 to 0.12)	986 (5)	0.001	Moderate/high
Mortality	101.14/1,000 population	120.69/1,000 population	0.02 (−0.01 to 0.05)	1,046 (5)	0.22^a^	Low

a Downgraded once because of large confidence intervals of the source studies.

nCPAP: nasal CPAP.

## Discussion

In this scoping review, key findings were moderate certainty of evidence to support the use of less invasive methods of SRT to limit the need for IMV. LISA/MIST was preferable to INSURE. Poractant alfa is conditionally recommended in preference to beractant for its superiority in limiting the need for invasive ventilation and reducing mortality. No recommendations could be made about the potential for different interventions to reduce the burden of BPD, nor for the superiority of a particular mode of NIV other than CPAP. While not within the scope of this review, data on medium- and longer-term outcomes, as well as cost-effectiveness, were sparse. This review highlighted large gaps in data from LMIC countries on surfactant practices, the gaps possibly due to unavailability of surfactant, lack of basic support in the management of RDS, or lack of reporting of use and outcomes. Africa, South America, and Indonesia were especially poorly represented (6).

The noted knowledge gaps include very limited reporting of local availability of surfactant in most LMICs; best clinical parameters for reliably indicating surfactant need; most appropriate method and timing of delivery of SRT, along with procedural sedation (taking into consideration local practice and constraints); as well as SRT impact on long-term outcomes.

The number of studies comparing INSURE with less invasive methods of SRT has increased, but most studies suggest that INSURE remains the predominant method of SRT administration, particularly in RRS. This has been attributed partly to the lag period of technology transfer from LMICs to HICs, with the cost of technology, lack of knowledge, and inadequate support systems being contributing factors to this lag period (14).

In keeping with guidelines from HICs (104), less invasive SRT administration demonstrated superiority over INSURE in mitigating the need for ventilation. A Cochrane review (105) published in 2021 concluded that SRT via a thin catheter (vs. via the ETT) has a similar rate of adverse effects and yet is associated with a lower intubation rate in the first 72 h, a reduced incidence of major complications and in-hospital mortality, and a reduced risk of death or BPD. However, some experts (106) in HICs suggest that the clinical potential of LISA techniques is overstated and urge the conduct of further high-quality studies.

Limited published data from LMICs on other “less invasive” methods of SRT administration—such as via laryngeal or supraglottic airways (SALSA), or aerosolization—were noted. Because of the lack of expertise in laryngoscopy in many LMICs, surfactant administration through SALSA is a promising method of SRT (107). It is relatively easy to insert a supraglottic airway device (SAD) into the mouth and advance it until it meets resistance. However, minimal published evidence involving SALSA in LMICs was available for inclusion in this study, and the quality of evidence was low (108). An important recent development has been the manufacture of smaller SADs that can be used in infants weighing less than 1,000 g (109). Aerosolization of surfactant is the least invasive method of delivering it, requiring no airway manipulation. Several studies have shown that although it is considered safe, the efficacy of aerosolization is still inconclusive (110).

Poractant was found to be superior to beractant for reducing mortality and progression to IMV. This is in keeping with a Cochrane review (111), which demonstrated an increased risk of in-hospital mortality, oxygen requirement at 36 weeks' gestational age, and PDA requiring treatment in infants treated with beractant vs. poractant. However, a lack of dose-equivalent comparison groups of appropriate sample size was a potential reason for differences. However, Boshoff Coyles (112) demonstrated no significant differences in outcomes between two groups of infants given these different surfactants at comparable dosages. Further high-quality as well as cost-effectiveness studies are needed.

With increasing survival at younger gestational ages, the importance of subsequent morbidity is paramount. The current review found that neither the technique of administration, the type of surfactant used, nor the type of respiratory support (NIV vs. CPAP) had any significant effect on the outcome of BPD, despite all three being associated with a reduced need for invasive ventilation, which is a proxy for the likelihood of long-term morbidity. The need for invasive ventilation may be an inappropriate proxy for this outcome, as it is only one of many factors contributing to BPD development, which is known to occur in infants who have never been invasively ventilated (106).

The use of NIV other than CPAP was found to be superior to CPAP with regard to the need for IMV, yet no effect on mortality was shown. This contrasts with the results of an RCT published in 2015 (113), which found that non-invasive neurally adjusted ventilatory assist (NIV NAVA) had no significant effect on oxygen requirements or the need for invasive ventilation in preterm newborns compared with CPAP. A RCT (91) that compared HFNC vs. CPAP found that although INSURE failure was higher in the HFNC group, HFNC was easier to use for nurses, better tolerated for infants, and facilitated more attachment between infants and parents—considerations that may justify its use. A three-arm multicenter randomized controlled trial comparing HFNC vs. CPAP vs. NIPPV as primary respiratory support in infants ≥32 weeks GA is currently registered with the Chinese Clinical Trial Registry (114). However, high-tech NIV is unlikely to be available in many RRS, and availing CPAP—even in its most rudimentary forms—remains a priority (6, 9, 13, 15–19, 115–119).

The answer to a “one-size-fits-all-SRT-protocol-for-best-outcomes” remains elusive. This is true even in HICs. A recent publication, The Respiratory Distress Syndrome Neonatal Expert Taskforce (RDS-NExT) (118) initiative, convened a panel of experts from various regions of the United States to establish consensus on best clinical surfactant practices. One finding was the absence of standard practices in respiratory management and surfactant administration. This variability demonstrates a lack of consensus regarding how SRT is used in the neonatal setting even in HICs. Tailored SRT approaches should be based on the best local evidence. For example, in LMICs, a lack of routine early pregnancy ultrasound scans and high rates of intrauterine growth restriction diminish the utility of birthweight and GA estimates in SRT decision-making (119). Clinical parameters, then, should be prioritized, as is the case in most guidelines, including those from high-income settings.

The 2022 Update of the European Consensus Guidelines on the Management of Respiratory Distress Syndrome (104) advise that surfactant be given where there is worsening RDS with FiO_2_ >0.30 on CPAP pressures ≥6 cmH_2_O, or where LUS suggests surfactant deficiency, and that even lower FiO_2_ thresholds be considered for very immature infants. Variability in precise FiO_2_ or SPO_2_ cut-offs observed in studies may reflect resource constraints. The use of higher FiO_2_ or lower saturation target thresholds—as noted in some of the included studies—raises the question of whether “permissive hypoxemia” is clinically consequential, which is another research gap. On the other hand, the financial burden of administering surfactant at lower thresholds of FiO_2_ is an important consideration in LMICs, where the cost of surfactant is often borne by the families of the infants (29). The RDS-NExT study highlights that individual clinical parameters and available resources must always be considered in deciding when and how SRT is delivered (118).

The factor of timing of SRT may be especially important in RRS where mothers may have received little to no antenatal care or steroids or where infants are often “outborn” and require transport (sometimes over great distances) to health facilities. While a Cochrane (120) review found that early stabilization with CPAP and selective SRT (“rescue”) to infants requiring intubation yielded less risk of chronic lung disease or death, the trials included were mostly from HICs, thus limiting the generalizability of these findings. Prophylactic surfactant may be beneficial dependent on the setting. While the heterogeneity of timing among studies included in this review precluded meta-analysis, those with statistically significant findings tended to favor early over late administration. A lack of equipment and expertise outside of the NICU setting may mean that alternate measures of delivering SRT are needed.

Procedural sedation is an underexplored component of optimal SRT. Laryngoscopy and airway manipulation can precipitate dangerous physiological responses, including apnea, bradycardia, and laryngospasm, as well as discomfort in the absence of appropriate sedation (106). A balance of light sedation/analgesia is needed to mitigate these responses, while not impeding a quick extubation post procedure. Few studies included premedication practices, despite the recommendation by both American and European guidelines (121, 122) that sedation be provided for laryngoscopy. Considering that overly deep sedation creates the need for prolonged ventilation, while insufficient sedation may result in neurosensory impairment associated with early neonatal pain experiences (102), this is another important area of study for future research.

This research project used a mixed review methodology, which was planned *a priori* to include meta-analyses if sufficiently homogeneous data were available to pool results within specific research subquestions. The exclusion of non-English studies, which was pragmatically necessary for this unfunded study, is a substantial limitation of the review, as relevant studies from LMICs may have been omitted. There is potential bias implicit in the scoping review methodology, and given the limitations of selection bias and variable heterogeneity between studies in this scoping review, a collaborative LMIC multi-institutional research project may be an innovative research project to reduce heterogeneity and address gaps in data. We recommend prospective systematic reviews specific to each population intervention control outcome (PICO)-type subquestion to confirm efficacy and safety.

## Conclusion

In LMICs, where invasive mechanical ventilation is a scarce resource, LISA should be recommended in preference to INSURE. Poractant alfa (200 mg/kg) is also conditionally recommended in preference to beractant (100 mg/kg) for SRT, but regionally relevant cost- effectiveness studies are recommended to inform recommendations for implementation in LMICs. Observational studies regarding local practice and equipment and resource availability are needed to better describe current practice, shortfalls, and opportunities for strengthening SRT practices and improving outcomes. While more data have accumulated in the past decade, many gaps remain. In striving to achieve the SDG of fewer than 12 per 1,000 neonatal deaths, bundles of care from antenatal care and steroid coverage through to SRT and IMV must be strengthened.

## Data Availability

The raw data supporting the conclusions of this article will be made available by the authors without undue reservation.
