# Supplementary material for: A systematic scoping review of the use of surfactant replacement therapy for respiratory distress syndrome in preterm neonates in low- and middle-income countries
Source: Front Pediatr. 2025 Nov 7;13:1685625. doi: 10.3389/fped.2025.1685625 (PMC12634560; doi:10.3389/fped.2025.1685625)
Supplement: Supplementary file 1 [file Datasheet1.pdf]

Appendices

Appendix 1. Supplementary Tables

Table 1. Included studies (RCTs)

Author, year	Country	WBI group	Study type	Population	Intervention	Control	Primary Outcome
Halim, 2019	Pakistan	LMIC	RCT<br>MoA	(n = 100)<br>Inborn, ≤34w, spontaneously breathing + RDS	(n = 50) LISA, using 6Fr NG tube<br>+ nCPAP<br>(Survanta, 100mg/kg)	(n = 50) INSURE, 15-20 mins PPV	Need for MV
Gupta, 2020	India	LMIC	RCT<br>MoA	(n = 58)<br>28-34w + RDS	(n = 29) MIST, using 5Fr NG tube + nIPPV<br>(Curosurf, 200mg/kg)	(n = 29) INSURE, 'few mins' PPV	Need for MV in first 72 h of life
Mohammadizadeh, 2015	Iran	LMIC	RCT<br>MoA	(n = 38)<br>Inborn, ≤34w, BW≤ 1000-1800g, + RDS first hr of life + SRT within 30 mins of nCPAP	(n = 19) CATH, using 4Fr end hole feeding tube + nCPAP<br>(Curosurf, 200mg/kg)	(n = 19) INSURE, PPV continued for ≥1 min or until SpO2≥ 87%	Need for MV in first 72 h of life
Nayeri, 2014	Iran	LMIC	RCT<br>MoA	(n = 42)<br>≤28 w + minimal RDS symptoms or 28-35w + RDS or req	(n = 21) INSURE, MV weaned over 1 hour<br>(not specified)	(n = 21) CMV + surfactant via ETT, 'long-term' (several days) MV	Requiring MV on day 5 of life

Author, year	Country	WBI group	Study type	Population	Intervention	Control	Primary Outcome
				FiO2≥0.45			
Kanmaz, 2013	Turkey	UMIC	RCT MoA	(n = 200) Inborn, <32w + RDS	(n = 100) 'Take Care' using 5Fr NG tube + nCPAP (Curosurf, 100mg/kg)	(n = 100) INSURE, T-piece breaths during SRT, prompt extubation	Need for MV in first 72 h of life
Li, 2016	China	UMIC	RCT MoA	(n = 44) 27 - 31+6 w, + RDS grade I to II on CXR	(n = 22) LISA (Curosurf, dose not specified)	(n = 22) INSURE	Cerebral oxygen saturation
Okur, 2019	Turkey	UMIC	RCT MoA	(n = 14) 26-32w, BW≤1250g, + RDS	(n = 7) MIST, using 5Fr NG tube + nCPAP (Curosurf, 200mg/kg)	(n = 7) INSURE, T-piece breaths during SRT, prompt extubation	NIPE and PIPP indices (pain
Bao, 2015	China	UMIC	RCT MoA	(n = 90) 28-32w, + RDS, req SRT within 2 h of birth	(n = 47) LISA, using 16G, 130mm vascular catheter (16G Angiocath) + nCPAP (Curosurf, 200mg/kg)	(n = 43) INSURE, 'some brief MV,' ETT withdrawn 'as soon as clinically possible' post-SRT	Need for MV in first 72 h of life
Pareek, 2021	India	LMIC	RCT MoA	(n = 40) 28-36+6w, spontaneous breathing, +RDS within 24h of life,	(n = 20) LISA, using 5Fr feeding tube, NIV (NCPAP or NIPPV)	(n = 20) INSURE, SRT followed by PPV w T-piece; ETT removed thereafter	Need for MV in first 72 h of life

Author, year	Country	WBI group	Study type	Population	Intervention	Control	Primary Outcome
				meeting ≥2 criteria for SRT	(not specified)<br>(dose 100mg/kg)		
Jena, 2019	India	LMIC	RCT<br>MoA	(n = 350)<br><34w, + RDS, req SRT within first 6h of life	(n = 175)<br>SurE, using 16G angiocath or 6Fr feeding tube<br>(Neosurf [bovine]) (135mg/kg)	(n = 175)<br>InSurE, SRT followed by PPV w T-piece; ETT removed thereafter	Need for MV in first 72 h of life
Barbosa, 2017	Brazil	UMIC	RCT<br>MoA	(n = 48)<br>28-35w, BW ≥1000g, <8h of age, req. nCPAP, SAS >4 and/or RR >60bpm and/or FiO2 ≥0.40 to maintain SpO2 91-95%, +RDS	(n = 26)<br>LMA, using ProSeal size one LMA, insertion classical technique, connected to self-inflated bag (PPV), 6-F catheter (modified length) conduit for SRT through LMA. LMA removed >SRT --> nCPAP<br>(Curosurf, 200mg/kg)	(n = 22)<br>Oral ETT, +premed, verification tube placement by CXR prior to SRT. SRT followed by conventional MV, extubated 'as soon as possible' (PIP <20 cmH2O, RR ≤30 bpm, and FiO2 < 0.4)	FiO2 ≤ 0.30 after SRT
Sabzehei, 2022	Iran	LMIC	RCT<br>MoA	(n = 112)<br>28-36w, +RDS	(n = 56)<br>MIST, using 5Fr feeding tube, nCPAP<br>(Curosurf, 200mg/kg)	(n = 56)<br>ETT, SRT via feeding tube through ETT, thereafter 'received PPV' and extubated to NCPAP	Need for MV in first 72 h of life
Yang, 2020	China	UMIC	RCT<br>MoA	(n = 97)<br>32-36+6w, req nCPAP within 12h of	(n = 47)<br>LISA, using 6Fr gastric tube (external diameter	(n = 50)<br>ETT + PPV, PPV continued for 3 mins >	Need for MV in first 72 h of life

Author, year	Country	WBI group	Study type	Population	Intervention	Control	Primary Outcome
				birth, +RDS, nCPAP pressure >6 cmH2O & FiO2 >0.40	2mm), nCPAP throughout (Curosurf, 200mg/kg)	SRT, thereafter extubated to nCPAP	
Han, 2020	China	UMIC	RCT MoA	(n = 298) 25-31+6w, RDS on nCPAP, FiO2 >0.40 for SpO2 >85%, req SRT within 6h of life	(n = 151) MISA, using 5F end hole gastric tube catheter, nCPAP (calf pulmonary surfactant, 100mg/kg)	(n = 147) EISA, only if nCPAP failure, intubation w ETT, SRT via ETT	Difference in the morbidity of BPD SA and EISA groups at 36w CGA
Anand, 2022	India	LMIC	RCT MoA	(n = 150) 26-34w, + RDS req SRT within 6h of life, on nCPAP & stable	(n = 74) LISA, 8Fr feeding tube, nCPAP	(n = 76) INSURE, ETT inserted, SRT given via feeding tube passed through ETT, 4 boluses w PPV > each bolus, ETT removed immediately post-SRT --> nCPAP	Difference in total duration (hours) ory support between LISA and
Mishra, 2022	India	LMIC	RCT MoA	(n = 150) 28-36w, spontaneously breathing, +RDS	(n = 75) 5Fr feeding tube, NIPPV throughout (Beractant, 100mg/kg)	(n = 75) InSurE, SRT administered through 5Fr feeding tube passed through ETT, followed by PPV w self-inflating bag. Extubated 'after a few minutes' -->	Need for MV in first 72 h of life

Author, year	Country	WBI group	Study type	Population	Intervention	Control	Primary Outcome
						NIPPV	
Sadeghnia, 2022	Iran	LMIC	RCT<br><br>MoA	(n = 50)<br><br>28-32w, req SRT due to RDS, within first 2h of life	(n = 25)<br><br>Aerosolization via mesh nebulizer (Aerogen Solo©) attached to inspiration arm w T-piece x 30 mins, nCPAP throughout<br><br>(Survanta, dose/weight)	(n = 25)<br><br>INSURE, SRT via ETT in 4 aliquots, each followed with PPV ≥1 minute. Timing of extubation not specified	Primary outcome not specified.<br><br>Need for MV
Babaei, 2019	Iran	LMIC	RCT<br><br>SRT aug.	(n = 80)<br><br>28-34w, w RDS, BW > 1000g, negative blood culture	(n = 40)<br><br>Surfactant (Chiesi, Italy), 2.5ml/kg<br><br>Ventolin 0.2mg/kg given with surfactant<br><br>Nil further detail on method of administration.	(n = 40)<br><br>Surfactant (Chiesi, Italy), 2.5ml/kg<br><br>Normal saline 0.5ml/kg given with surfactant<br><br>Nil further detail on method of administration.	Multiple "primary outcomes;"<br>eed for MV'
Gharehbaghi, 2021	Iran	LMIC	RCT<br><br>SRT aug	(n = 128)<br><br><30w, BW < 1500g, RDS req SRT	(n = 64)<br><br>INSURE, intratracheal instillation Budesonide 0.25mg/kg + surfactant<br><br>Extubated to nCPAP once spontaneous respirations resumed & HR & SpO2 adequate<br><br>Curosurf, 200mg/kg	(n = 64)<br><br>INSURE, intratracheal instillation SRT only<br><br>Extubated to nCPAP once spontaneous respirations resumed & HR & SpO2 adequate	Incidence of BPD

Author, year	Country	WBI group	Study type	Population	Intervention	Control	Primary Outcome
Celik, 2018	Turkey	UMIC	RCT<br>SRT aug	(n = 40)<br><37w (26 - 36w), +RDS	(n = 20)<br>INSURE + inhaled salbutamol 0.15mg/kg stat by micropump nebulizer 10 minutes prior to first SRT<br>Curosurf, 200mg/kg	(n = 20)<br>INSURE + inhaled normal saline solution 0.30ml/kg stat by micropump nebulizer 10 minutes prior to first SRT	Multiple parameters reflecting salbutamol therapy,' including espiratory support
Dehdashtian, 2016	Iran	LMIC	RCT<br>SRT aug	(n = 48)<br><34w, BW > 1000g, RDS + neg blood culture	(n = 24)<br>INSURE, within 2h of birth + intratracheal salbutamol 0.2mg/kg immediately after SRT via same intratracheal tube<br>Curosurf, 200mg/kg	(n = 24)<br>INSURE, within 2h of birth + intratracheal normal saline 0.5ml/kg immediately after SRT via same intratracheal tube	Need for MV within 72h of
Kandraju, 2013	India	LMIC	RCT<br>ToA	(n = 153)<br>28-33+6w, RDS within 2h of birth, commenced on nCPAP first line	(n = 74)<br>Early - received SRT immediately after enrollment, via INSURE method. PPV > each aliquot (2-3), extubated back to nCPAP once SpO2 maintained 87-93%<br>Curosurf or Survanta, 100mg/kg	(n = 74)<br>Late - received SRT if/when required FiO2 > 0.50 beyond 2h of life, via INSURE method. PPV > each aliquot (2-3), extubated back to nCPAP once SpO2 maintained 87-93%	Need for MV in the first 7 days of
Rong, 2019	China	UMIC	RCT	(n = 305)	(n = 154)	(n = 151)	Need for MV in first 72 h of life

Author, year	Country	WBI group	Study type	Population	Intervention	Control	Primary Outcome
			ToA	26-32+6w, RDS req nCPAP shortly > birth	Early - as soon as possible once RDS 'criteria' met<br>INSURE - PPV > SRT, extubated to nCPAP once SpO2 maintained<br>Calsurf 100mg/kg	Late - only once CXR displayed RDS (usually ≥ 1-2h for X-ray to be done)<br>INSURE - PPV > SRT, extubated to nCPAP once SpO2 maintained	
Okulu, 2015	Turkey	UMIC	RCT<br>ToA	(n = 80)<br><30w, inborn	(n = 40)<br>Early - as soon as possible after birth (with first breath)<br>INSURE - extubated to nCPAP > completion SRT<br>Curosurf 100mg/kg	(n = 40)<br>Late - 15 m > first breath<br>INSURE - extubated to nCPAP > completion SRT	Need for MV in first 72 h of life
Kong, 2016	China	UMIC	RCT<br>ToA	(n = 207)<br>25-27+6w; and 28-31+6 with ≥3: (1) mothers with diabetes or gestational diabetes; (2) male infants; (3) multiple births; (4) mothers without antenatal steroid or who received insufficient dose; (5) emergency intubation requirement after	(n = 116)<br>Prophylactic - within 30m of birth<br>INSURE - intubated in delivery room, +SRT within 30m of birth (ideally 15m)<br>Ventilation ≥ 1-3h >SRT<br>Calsurf, 70mg/kg	(n = 91)<br>Early - received SRT for established RDS within 30m - 2h post birth<br>Indications: apnea, req suppl O2, significant RD, req PPV, characteristic CXR<br>INSURE + ventilation ≥ 1-3h >SRT	Incidence of RDS

Author, year	Country	WBI group	Study type	Population	Intervention	Control	Primary Outcome
				birth; and (6) severe respiratory distress shortly after birth highly suggestive of RDS			
Yilmaz, 2021	Turkey	UMIC	RCT<br>STs	(n = 193)<br>22-34w, +RDS admitted to NICU	(n = 77)<br>Beractant 100mg/kg<br>INSURE - extubated as soon as possible depending on resp status	(n = 59)<br>Poractant alfa, 200mg/kg<br>INSURE - extubated as soon as possible depending on resp status<br>(n = 57)<br>Calfactant, 100mg/kg<br>INSURE - extubated as soon as possible depending on resp status	Duration of MV (days)
Jain, 2019	India	LMIC	RCT<br>STs	(n = 98)<br>26-32w, +RDS within 6h of birth, fulfilled criteria for SRT within 24h birth	(n = 52)<br>Goat lung surfactant extract (GLSE)<br>INSURE, 4 aliquots, extubated CPAP > SRT if adeq resp effort & HD stable	(n = 46)<br>Beractant<br>INSURE, 4 aliquots, extubated CPAP > SRT if adeq resp effort & HD stable	Composite of death or [illegible]monary dysplasia
Dizdar, 2012	Turkey	UMIC	RCT<br>STs	(n = 126)<br><37w, + RDS within 6h birth	(n = 61)<br>Poractant alfa, 200mg/kg<br>No method specified	(n = 65)<br>Beractant, 100mg/kg<br>No method specified	FiO2 at 24h post-gestation
Bozdag, 2015	Turkey	UMIC	RCT	(n = 42)	(n = 21)	(n = 21)	Change in respiratory status

Author, year	Country	WBI group	Study type	Population	Intervention	Control	Primary Outcome
			STs in Pulm Haem	<32w or BW <1500g, + pulmonary haemorrhage within first 2w life	Poractant alfa, 100mg/kg<br>Method not specified (all already intubated)<br>Given > 2nd h of pulm haem	Beractant, 100mg/kg<br>Method not specified (all already intubated)<br>Given > 2nd h of pulm haem	RT (reflected by oxygenation index<br>variables)
Najafian, 2016	Iran	LMIC	RCT<br>STs	(n = 112)<br>BW >750g, GA <35w, +RDS, inborn, SpO2 85-96%, age ≤6h at time of randomization	(n = 56)<br>Curosurf, 100mg/kg<br>INSURE - extubation immed > SRT to nCPAP	(n = 56)<br>Survanta, 100mg/kg<br>INSURE - extubation immed > SRT to nCPAP	Need for MV
Bozkaya, 2021	Turkey	UMIC	RCT<br>STs	(n = 73)<br>26 - 31+6w, adm NICU w RDS, SRT within 6h life	(n = 37)<br>Poractant alfa, 200mg/kg<br>INSURE	(n = 36)<br>Beractant, 100mg/kg<br>INSURE	Evaluation of LUS scores and<br>r administration of different surf<br>s
Mirzarahimi, 2018	Iran	LMIC	RCT<br>STs	(n = 150)<br><37w, +'HMD'	(n = 75)<br>Curosurf, 100mg/kg<br>Via ETT	(n = 75)<br>Survanta, 100mg/kg<br>Via ETT	Duration of MV (hours)
Mussavi, 2016	Iran	LMIC	RCT<br>STs	(n = 165)<br>≤ 37 weeks, +RDS, adm NICU	(n = 54)<br>Alveofact, 100mg/kg<br>INSURE; >SRT, manual PPV 3-5 min; extubated once satisfactory resp drive &<br><br>(n = 62)<br>Curosurf, 200mg/kg<br>INSURE; >SRT, manual PPV 3-5 min; extubated once satisfactory	(n = 49)<br>Survanta, 100mg/kg<br>INSURE; >SRT, manual PPV 3-5 min;	CPAP failure;<br>Ventilator dependence until 7d

**Author, year**	**Country**	**WBI group**	**Study type**	**Population**	**Intervention**	**Control**	**Primary Outcome**
					oxygenation<br>resp drive & oxygenation	extubated once satisfactory resp drive & oxygenation	
Macooie, 2018	Iran	LMIC	RCT<br>STs	(n = 100)<br>Infants w RDS hospitalised in study hosp	(n = 50)<br>Bovine lipid extract (BLES), 100mg/kg<br>Method not specified	(n = 50)<br>Survanta, 100mg/kg<br>Method not specified	Primary outcome not specified<br>(incl continuation of MV - inv &
Karadag, 2015	Turkey	UMIC	RCT<br>STs	(n = 92)<br>≤32 w, +RDS, req MV, age at randomization of 2h post delivery	(n = 46)<br>Beractant, 100mg/kg<br>INSURE, extub to nCPAP as early as possible	(n = 46)<br>Poractant alfa, 200mg/kg<br>INSURE, extub to nCPAP as early as possible	Perfusion index (PI) variability after SRT
Sarokolai, 2018	Iran	LMIC	RCT<br>STs	(n = 240)<br>≤ 34 w, +RDS	(n = 120)<br>Curosurf, 200mg/kg<br>INSURE	(n = 120)<br>Bovine lipid extract (BLES), 5mL/kg. INSURE	Primary outcome not specified;<br>n index 'main' outcome)
Gharehbaghi, 2010	Iran	LMIC	RCT<br>(STs)	(n = 150)<br>'Preterm,' + RDS req SRT	(n = 79)<br>Poractant alfa, 200mg/kg<br>INSURE	(n = 71)<br>Beractant, 100mg/kg<br>INSURE	Remained without ventilator<br>ugh 7 days of age
Rebello, 2014	Brazil	UMIC	RCT<br>(STs)	(n = 327)<br>BW 501-1500g, age ≤24 h of life, req MV + PaO2/FiO2 ratio	(n = 154)<br>Butantan, 100mg/kg<br>INSURE	(n = 173)<br>Curosurf or Survanta, 100mg/kg	Mortality at 72 h; and mortality at

Author, year	Country	WBI group	Study type	Population	Intervention	Control	Primary Outcome
				≤175, + RDS		INSURE	
Terek, 2014	Turkey	UMIC	RCT (STs)	(n = 48)<br>Study group (n = 30): 26-36w, early SRT for RDS, in study NICU	(n = 30)<br>Poractant alfa, 200mg/kg; or beractant 100mg/kg<br>INSURE, SRT (within 2h of birth) followed by 2 m manual PPV, remained intubated therafter and weaned	(n = 18)<br>Control group (n = 18): healthy, preterm, not req O2 or CPAP	Perfusion index, transcutaneous (TCO) changes, oxygenation & arameters
Kadivar, 2016	Iran	LMIC	RCT (IRS)	(n = 54)<br>28-34w, + RDS req SRT within 48 h after birth, extub within 1h > SRT	(n = 27)<br>NCPAP group<br>Survanta, 4ml/kg<br>INSURE	(n = 27)<br>HFNC group<br>Survanta, 4ml/kg<br>INSURE	Not requiring MV for at least 72 h
Malakian, 2021	Iran	LMIC	RCT (IRS)	(n = 148)<br>28-34w, + RDS, + Silverman-Anderson score 6 or 7 during first 6 h > birth	(n = 74)<br>DUOPAP group<br>Survanta, 100mg/kg<br>INSURE --> extub to same NIV used prior to INSURE	(n = 74)<br>NCPAP group<br>Survanta, 100mg/kg<br>INSURE --> extub to same NIV used prior to INSURE	Need for MV within first 72 h of
Meneses, 2011	Brazil	UMIC	RCT (IRS)	(n = 200)<br>26-33+6w, +RDS	(n = 100)<br>NCPAP group<br>Curosurf, 100mg/kg<br>INSURE --> extub to	(n = 100)<br>NIPPV group<br>Curosurf, 100mg/kg<br>INSURE --> extub to	Need for MV in first 72 h of life

Author, year	Country	WBI group	Study type	Population	Intervention	Control	Primary Outcome
					allocated mode	allocated mode	
Oncel, 2016	Turkey	UMIC	RCT<br>(IRS)	(n = 200)<br>26-32w, +RDS	(n = 100)<br>nCPAP group<br>MIST approach - gastric tube intratracheal; INSURE (ETT) if already intubated<br>Poractant alfa, 100mg/kg	(n = 100)<br>nIPPV group<br>MIST approach - gastric tube intratracheal; INSURE (ETT) if already intubated<br>Poractant alfa, 100mg/kg	Need for MV in first 72 h of life
Chen, 2015	China	UMIC	RCT<br>(IRS)	(n = 286 [143 pairs])<br>28-36w, twins, +RDS	(n = 143)<br>nIPPV group<br>Curosurf, 100mg/kg<br>INSURE	(n = 143)<br>nCPAP group<br>Curosurf, 100mg/kg<br>INSURE	Need for MV
Zhu, 2017	China	UMIC	RCT<br>(PRS)	(n = 76)<br>28-34w, moderate-severe RDS (Silverman score>6 within 1st h life)	(n = 37)<br>NHFOV<br>Curosurf, 200mg/kg<br>INSURE	(n =39)<br>nCPAP<br>Curosurf, 200mg/kg<br>INSURE	Need for MV
Shokouhi, 2019	Iran	LMIC	RCT<br>(PRS)	(n = 60)<br>28-36w, +RDS, NICU admission length <24h, FiO2 req > 0.40, 5-min Apgar score >5	(n = 30)<br>HFNC<br>Curosurf, 200mg/kg<br>INSURE	(n = 30)<br>nCPAP<br>Curosurf, 200mg/kg<br>INSURE	Primary outcome not specified<br>tion of resp support, requirement of<br>ed)
Akbarian-rad, 2018	Iran	LMIC	RCT	(n = 64)	(n = 32 - 2) (2 excluded	(n = 32)	SpO2 values, oxygen need at 6,

Author, year	Country	WBI group	Study type	Population	Intervention	Control	Primary Outcome
			(PRS)	27-34w, req SRT during first 2h > birth for RDS	post-randomization)<br>HHHFNC group<br>Curosurf, 200mg/kg<br>INSURE, extubated ≤30 mins > SRT if acceptable ABG	nCPAP group<br>Curosurf, 200mg/kg<br>INSURE, extubated ≤30 mins > SRT if acceptable ABG	post SRT
Pan, 2021	China	UMIC	RCT<br>(PRS)	(n = 284)<br>BW < 1500g, inborn, + RDS, > SRT	(n = 140)<br>nCPAP<br>INSURE, extubated if SpO2 90-95% in FiO2 ≤0.25 w no incr RD and/or apnea	(n = 144)<br>BiPAP<br>INSURE, extubated if SpO2 90-95% in FiO2 ≤0.25 w no incr RD and/or apnea	Need for MV in first 72 h of life
Karadag, 2016	Turkey	UMIC	RCT	(n = 125)<br>26-32w, BW 600-1500g, +RDS within 3h of life, inborn, req FiO2 ≥0.4 to maintain SpO2 88-96%	(n = 42) Group I<br>4 positions, as suggested by manufacturer<br>Beractant 100mg/kg<br>INSURE<br>Extubated if requiring FiO2 <0.4	(n = 42) Group II<br>2 positions<br>Beractant 100mg/kg<br>INSURE<br>Extubated if requiring FiO2 <0.4<br><br>(n = 41) Group III<br>Neutral position<br>Beractant 100mg/kg<br>INSURE<br>Extubated if requiring FiO2 <0.4	FiO2 and SpO2 before, during rfactant administration
Nakhshab, 2015	Iran	LMIC	RCT	(n = 60)<br>27-34+6w, RDS within first 6h of life,	(n = 30)<br>INSURE	(n = 30)<br>NCPAP alone	Need for MV

Author, year	Country	WBI group	Study type	Population	Intervention	Control	Primary Outcome
				RDS score >5, inborn	Survanta 4mL/kg<br>Manual PPV using Neopuff > each aliquot; extubated as soon as stable (within 1 h)	Received rescue surfactant via INSURE method<br>Survanta 4mL/kg<br>Extubated as soon as stable (within 1 h)	
Sk, 2022	India	LMIC	RCT	(n = 34)<br>28-33+6w, RDS	(n = 17)<br>LISA + Fentanyl<br>5Fr feeding tube; withdrawn > SRT. nCPAP throughout	(n = 17)<br>LISA only<br>5Fr feeding tube; withdrawn > SRT. nCPAP throughout	Proportion of infants with revised nfant pain profile (R-PIPP) score ≤ e LISA procedure
Sabzehei, 2022	Iran	LMIC	RCT<br>(IRS & PRS)	(n = 95)<br>28-36w, +RDS w Downes score≥3 at birth, not requiring DR MV, inborn	(n = 48)<br>nIPPV group<br>Both given rescue SRT as per criteria. Poractant alfa, 200mg/kg, via LISA.	(n = 47)<br>nCPAP group	Need for MV in first 72 h of life

*Surfactant doses refer to initial dose

Supplementary Table 2: Included studies (Observational)

Author	Country	WBI group	Methodology	Population	Intervention	Control	Primary Outcome(s)
Vardar, 2020	Turkey	UMIC	Diagnostic accuracy; prospective & double blind cohort	(n = 45)<br><34w, +RDS req SRT	Lung ultrasound	CXR	(1) Correlation between LUS score and severity of RDS comparing w CXR<br>(2) Predict need for SRT using LUS score in preterm neonates
Afjeh, 2017	Iran	LMIC	Prospective inception cohort<br>(IRS)	(n = 499)<br>In-born VLBW neonates + RDS (outborns if admitted <24h old)	3 groups compared:<br>Group I: O2 therapy via blender ± oxyhood<br>Group II: nCPAP/nIPPV<br>Group III: MV ± SRT		Not differentiated.<br>Incl: Failure & success initial resp. Support strategy (IRS) & mortality
Azzabi, 2016	Tunisia	LMIC	Prospective cohort analysis (RFs INSURE failure)	(n = 40)<br>27-35w, +RDS	All received SRT via INSURE method using porcine surfactant, 100-200mg/kg		INSURE failure
Nanda, 2020	India	LMIC	Prospective, score derivation & validation<br>(fx predicting need of SRT)	(n = 209)<br>Inborn, singleton, 26+0 - 34+6w, +RDS	All received SRT		Significant variables established by multivariate regression analysis
Dizdar, 2011	Turkey	UMIC	Prospective cohort	(n = 69)	All received poractant alfa, 200mg/kg		Total antioxidant

Author	Country	WBI group	Methodology	Population	Intervention	Control	Primary Outcome(s)
			(oxidant/antioxidant status)	<37w, +RDS & received SRT within 12h after birth	via intratracheal method (INSURE) within 12 h of birth		capacity (TAC) measurement & total antioxidant status (TOS) measurement pre-SRT & 48h thereafter
Tagare, 2014	India	LMIC	Prospective analytical (outcome & feasibility INSURE)	(n = 28) <37w, on nCPAP ≥ 30 mins with FiO2 requirement > 0.40 within first 6 h of life	All received bovine surfactant, 4mL/kg via INSURE		INSURE success
Ngo, 2020	Vietnam	LMIC	Prospective cohort (MoA)	(n = 106) 26-32w, +RDS, req nCPAP within first 6h life + SRT	LISA (n = 53) Size 6F gastric tube Type not spec, 200mg/kg	INSURE (n = 53) PPV continued x 3 mins > SRT. Extub to nCPAP	Not differentiated Incl: MV req within 72h
Mahmoud, 2019	Egypt	LMIC	Case-control (study sample compared to historical cohort) (IRS + PRS)	(n = 82) 28-34, +RDS, admitted to NICU	nIPPV (n = 41) Both received Survanta, 100mg/kg, in the event of resp support strategy failure, via Hobart instillation (5F rigid sterile arterial umbilical catheter; i.e. LISA)	nCPAP (n = 41)	Not differentiated Incl: req MV, BPD, mortality
Danaei, 2017	Iran	LMIC	Cross-sectional	(n = 192)	All received Survanta, 100mg/kg via INSURE with manual ventilation PRN		Not differentiated

Author	Country	WBI group	Methodology	Population	Intervention	Control	Primary Outcome(s)
			(RFs INSURE failure)	26-36w, BW 500-3500g, hosp up to 48h > birth, +RDS	until 'acceptable' HR & SpO2 --> thereafter, extubated to nCPAP		Incl: neonatal morbidity (incl. Chronic lung disease)
Eras, 2014	Turkey	UMIC	Prospective, longitudinal, single-center, cohort study w two arms<br>First arm: STs<br>2nd arm: Neurodev. Outcomes	(n = 215)<br>≤32 weeks, +RDS<br>28 lost to follow up	Poractant alfa (n = 98)	Beractant (n = 89)	Neurodevelopmental impairment as defined by ≥ 1 of the following: (1) cerebral palsy w functional deficits, (2) bilateral hearing loss and/or blindess, and (3) MDI or PDI < 70 on BSID II
Fallahi, 2020	Iran	LMIC	Prospective cohort (IRS)	(n = 163), VLBW, admitted to study centre NICU, +RDS	(n = 82) Non-invasive group (oxyhood, room air, nCPAP & nIPPV)	(n = 81) Invasive group (INSURE, MV with or without SRT)	Not differentiated<br>Incl: survival rate
Duman, 2016	Turkey	UMIC	Prospective cohort, single-center<br>(ToA; method IRS)	(n = 65), 24-31+6w, postnatal age within 60 min, suppl. O2 req w	(n = 30) nIPPV + surfactant	(n = 29) nIPPV	Requiring MV within 72 h of enrollment into study

Author	Country	WBI group	Methodology	Population	Intervention	Control	Primary Outcome(s)
				SAS >3	Both groups given SRT if indication criteria met (ie. 2nd dose in intvn group). Poractant alfa, 200mg/kg via INSURE. PPV x 1 min --> extub to nCPAP		
Khan, 2015	Pakistan	LMIC	Prospective cross-sectional	(n = 52), ≤37w, + RDS within 12 h of birth, req SRT	All received Survanta, 100mg/kg via “intra-tracheal” route		Not differentiated<br>Incl: extubation rate within 48h, BPD, mortality
Vamseedharm 2015	India	LMIC	Prospective, single-center cohort<br>(ToA)	(n = 122)<br><34w, + RDS	Grp A: SRT within 12 h of birth<br>Grp B: SRT within 12-24h of birth	Grp C: SRT between 24-72h > birth<br>Grp D: No SRT received.	Not differentated<br>Incl: Mortality
Okwonkwo, 201	Nigeria	LMIC	Two-phase cross-sectional: (1) respondent-administered structured questionnaire; (2) SRT audit single-center using database<br>(extent of & benefits SRT)	Phase 1: (n = 237): HCWs working in tertiary & secondary public & private paediatric/neonatal unit (consenting PANCONF attendees)	Phase 2: (n =1124)<br>- preterm (<36w), n = 509<br>- received SRT, n = 15		Phase 1: SRT service availability<br>Phase 2: Mortality

Author	Country	WBI group	Methodology	Population	Intervention	Control	Primary Outcome(s)
Awaysheh, 2019	Jordan	UMIC	Prospective cohort (RFs INSURE failure)	(n = 63), <37w, + spontaneously breathing, eligible for SRT acc to unit guidelines	All given natural surfactant 3mL/kg via INSURE		Not differentiated<br>INSURE failure (ie. Req MV ≥72 h
You, 2021	China	UMIC	Prospective cohort with control group<br>(effect SRT)	(n = 216), 28-34w, singleton, no antenatal steroids, complete clinical data	nCPAP + SRT (n=114)<br>SRT via INSURE	nCPAP alone (n=102)	Multiple, including Incidence of neonatal RDS, requirement 2nd dose SRT, requirement of iMV, duration FiO2 & hospital stay
Singh, 2011	Mumbai	LMIC	Prospective cohort, before-and-after<br>(prophylactic SRT)	(n = 125), <34w	(n = 25), prophylactic SRT<br>100mg/kg INSURE 'shortly after birth'	(n = 100), rescue SRT<br>100mg/kg INSURE, as per unit protocol	Not differentiated<br>Incl: required iMV, chronic lung disease, mortality
Abdallah, 2023	Tanzania	LMIC	Prospective (prevalence CPAP failure)	(n = 74), <34w & BW 800-1500g, commenced on CPAP first 24h of life for RDS	Rescue surfactant given pending availability and parents' ability to pay for it. INSURE.		CPAP success or failure at 72h of age
Lategan, 2022	South	UMIC	Prospective, cross-	(n = 552), BW	Rescue surfactant given as per unit		Not differentiated

Author	Country	WBI group	Methodology	Population	Intervention	Control	Primary Outcome(s)
	Africa		sectional descriptive	<1801g, adm to one of 2 study centres (GSH or MMH)	protocol. Bovine surfactant, 100mg/kg via LISA.		
Buyutiryaki, 201	Turkey	UMIC	Retrospective cohort (folder review)<br>(MoA)	(n = 383), 25+0 - 29+6w, administered SRT either via LISA or INSURE	LISA-treated (n = 205), poractant-alfa, 200mg/kg. 5F, flexible, sterile NGT	INSURE-treated (n = 178), poractant-alfa, 200mg/kg. 'Prompt' extubated to NIV	Requiring MV within first 72 h of life
Ballot, 2015	South Africa	UMIC	Retrospective review (database)<br>(M&M VLBW infants in two time periods)	Overall, n = 1025<br>2013, n = 562<br>2006/7, n = 463<br>Both: BW 500-1500g, admitted to study centre within 48h life	Rescue SRT via INSURE, and nCPAP, provided as first-line therapy in all VLBW infants >750g at birth w RDS, in respiratory failure.		Survival to discharge
Buyutiryaki, 202	Turkey	UMIC	Retrospective folder review (IRS: nCPAP, BiPAP & nIPPV)	(n = 409), 26-30w, admitted to study centre NICU, +RDS, req nCPAP, BiPAP or nIPPV as initial resp support	nCPAP, (n = 221) | BiPAP, (n = 101) | nIPPV, (n = 97)<br>All received rescue SRT, poractant alfa 200mg/kg via LISA as per protocol		Failure of NIV within first 72 h of life
Şimşek, 2020	Turkey	UMIC	Retrospective folder review	(n = 200), ≤28w, +RDS, inborn	Poractant alfa, (n =112),	Beractant, (n = 88), 100m/kg. LISA or	Mortality before discharge

Author	Country	WBI group	Methodology	Population	Intervention	Control	Primary Outcome(s)
			(STs)		200mg/kg. LISA or INSURE	INSURE	
Wang, 2015	China	UMIC	Retrospective analysis<br>(therapeutic efficacy SRT per GA band)	(n = 135), received SRT during study time period for RDS	GA<35w, n = 54<br>All meeting prescribed criteria given porcine SRT, 200mg/kg as per unit protocol.	GA>35w & <37w, n =35<br>GA≥ 37, n = 46	Not differentiated<br>Incl: need for intubation, survival rate
Silahli, 2020	Turkey	UMIC	Retrospective folder review	(n = 65), <33w, +RDS, received SRT	LISA (n = 35), poractant alfa, 200mg/kg. 5F or 6F catheter	INSURE (n = 30)<br>poractant alfa, 200mg/kg. 'Rapid' extub --> nCPAP	Multiple, including: need for IMV, BPD
Chen, 2018	China	UMIC	Retrospective folder review<br>(comparison efficacy SRT between male & female cohorts)	(n = 370), +RDS received SRT	Male, n = 245<br>Porcine, 50-240mg/kg via INSURE given to both as required per unit protocol, as soon as practicably possible.	Female, n = 125	Mortality
Canpolat, 2020	Turkey	UMIC	Retrospective folder review<br>(Timing SRT & development of PDA)	(n = 593), <30w, ≤1,500g, +SRT given	Early, n = 365	Late, n = 228	Incidence of patent ductus arteriosus (PDA)
Xuan, 2020	Vietnam	LMIC	Retrospective folder review	(n = 215), 26-34w, adm < 72 h of life			Not differentiated<br>Incl: BPD, rate of

Author	Country	WBI group	Methodology	Population	Intervention	Control	Primary Outcome(s)
							MV, mortality
Naseh, 2014	Iran	LMIC	Retrospective folder review	(n = 242), =RDS	All given beractant or poractant alfa, 100mg/kg via INSURE. Extubated immediately > SRT --> nCPAP		Not differentiated<br>Incl: required MV post-INSURE, mortality rate
Wang, 2012	China	UMIC	Retrospective folder review	(n = 6864), received resp support <24 h, onset of resp failure <7 d			Not differentiated<br>Incl: BPD, mortality, requirement of MV
Sun, 2013	China	UMIC	Retrospective folder review	(n = 26,460), RDS (n = 3747), admitted to 1 of 2 study centers, with or without RDS	<32w, n = 1922<br>32+0 - 33+6w, n = 892	34+0 - 36+6w, n = 657<br>37+0 - 42+0w, n = 276	Not differentiated<br>Incl: SRT, mortality
Ognean, 2016	Romania	UMIC (2020)	Retrospective cohort (Romanian National Registry for RDS patients)<br>(RFs INSURE failure)	(n = 637), ≤32w	5 groups: (1) INSURE, CPAP; (2) SRT + MV; (3) MV; (4) MV + CPAP; (5) without RDS (no resp support, no SRT)<br>INSURE group, n = 57<br>Poractant alpha, 100-200mg/kg, within 2 h of birth		INSURE Failure: need for MV within 72h > INSURE
Ferri, 2020	Brazil	UMIC	Retrospective analysis	(n = 605), all VLBW	Group 1 (single	Group 2 (multiple	Not differentiated

Author	Country	WBI group	Methodology	Population	Intervention	Control	Primary Outcome(s)
			of prospectively collected data (hospital database)	preterm infants registered in database between 01/2006 and 12/2015	dose SRT), n = 410)<br>Survanta, 100mg/kg when threshold criteria met. Intratracheal	doses), n = 195	Incl: mortality, requirement of MV
Dobryanskyy, 2022	Ukraine	LMIC	Single-center, retrospective cohort (ToA)	(n = 165), <32w, BW<1,500g, preceding treatment with CPAP after birth for RDS	Early rescue group + LISA, n = 52, porcine 200mg/kg, or bovine 100mg/kg, depending on availability	Late rescue group + INSURE, n = 113, porcine 200mg/kg, or bovine 100mg/kg, depending on availability	Incidence of CPAP failure*<br>*Need for intubation and MV during first 5 d of life
Lessa, 2018	Brazil	UMIC	Cross-sectional study, employing a research database	(n = 626), BW 500-1749g born during time period, +RDS	Surfactant	Without surfactant	Surfactant use up to 2 h of life = outcome variable
Hamilton, 2017	Jamaica	UMIC	Retrospective descriptive	(n = 144), all premature neonates adm to study NICU 2001-2011	SRT given (n = 75)<br>No protocol for SRT criteria, surf available but not readily accessible secondary to cost	Not given SRT (n = 69)	Not differentiated<br>Incl: Survival

Author	Country	WBI group	Methodology	Population	Intervention	Control	Primary Outcome(s)
Xu & Bao, 2022	China	UMIC	Single-centre, retrospective observational cohort<br>(MoA)	(n = 148), RDS w GA <32w & BW <1,500g, spont breathing & stable in nCPAP, req SRT within 2 h after birth	LISA, (n = 46)<br>Porcine, 200mg/kg	INSURE, (n = 102)<br>Porcine, 200mg/kg	Occurrence & severity of BPD at 36w PMA or discharge, mortality before discharge, combined incidence mortality and BPD, MV rate within first 72h > birth
Patel, 2022	India	LMIC	Single-centre, retrospective observational cohort<br>(STs)	(n = 100), +RDS of prematurity + received SRT	Natural, (n = 52), Survanta 100mg/kg, INSURE	Synthetic (n = 48)<br>Phospholipids 67.5mg/kg, INSURE	Incl: mortality, treatment cost
Liu & Deng, 202	China	UMIC	Single-centre, retrospective observational cohort<br>(ToA)	(n = 90), premature infants w NRDS admitted to study centre	Control, n = 30, no SRT given<br>Early, n = 30, within 1.5 - 4h of life<br>SRT given via INSURE to those eligible	Late, n = 30, within 6-23h of life	Not differentiated<br>Incl: Duration MV, number of days survived
Zhang, 2023	China	UMIC	Retrospective cohort<br>(MoA)	(n = 210), 28-32w, +NRDS, required NIV post-birth, no intubation prior to SRT	LISA (n = 107)<br>Bovine, 100mg/kg, within 1 h of birth, small suction tube	INSURE (n = 103)<br>Bovine, 100mg/kg, within 1 h of birth, ETT removed > SRT --> NIV	Incidence of BPD at 36w CGA<br>Incidence of other related complications (NEC, IVH, ROP, pulm

Author	Country	WBI group	Methodology	Population	Intervention	Control	Primary Outcome(s)
							haem, air leak)
Ingemyr, 2022	South Africa	UMIC	Retrospective audit of existing database	(n = 938), 500 - 1500g, born between study period, admitted to study centre			Not differentiated - assessment of factors influencing survival and short-term outcomes of VLBW infants
Yilmaz, 2023	Turkey	UMIC	Cohort study, comparing two cohorts	(n = 579), BW 401-1500g or GA 22-29w, inborn or adm within 28 d of birth & registered in VON database by study NICU	Period 1 (n = 288), 01/2005 - 12/2009	Period 2 (n = 291)<br>01/2010 - 12/2019	Not differentiated<br>Incl: BPD, survival, morbidity-free survival
Cokyaman, 202[illegible]	Turkey	UMIC	Retrospective folder review (BPD freq & RFs in VLBW)	(n = 872), all VLBW infants admitted to study center	BPD (+), n = 139	BPD (-), n = 551	Not differentiated
Crivceanscaia, 2017	Republic of Moldova	UMIC	Prospective descriptive (IRS + MoA)	(n = 750), RDS secondary surfactant deficit or congenital pneumonia, GA <34w	Inborn (level III), n = 476<br>Outborn (aerial transfer), n = 240	Inborn + SRT via LISA, n = 34	Not differentiated<br>Incl: subsequent respiratory support, mortality In NICU, chronic lung disease
Kirsten, 2012	South	UMIC	Prospective descriptive	(n = 309), inborn, ELBW (BW 500-	Infants ≥750g or GA >26w eligible for SRT if study threshold criteria met.		Survival to day 7

Author	Country	WBI group	Methodology	Population	Intervention	Control	Primary Outcome(s)
	Africa			1000g), ≥25w, adm to NNU of study centre	Curosurf, 100mg/kg via INSURE. Extubated immed > SRT --> nCPAP		
Oncel, 2020	Turkey	UMIC	Survey (knowledge, attitudes & practices re LISA/MIST approach)	(n = 87), representing 195/350 neonatologists from both academic/public and private hospitals who are members of the Turkish Neonatal Society			Not differentiated<br>Incl: method SRT administration, frequency of use, dose, need for intubation
Hashim, 2021	Iraq	UMIC	Cross-sectional<br>(RFs INSURE failure)	(n = 50), 28-32w, +RDS, SRT via INSURE	INSURE success group, n = 31	INSURE failure group, n = 19	Not differentiated<br>Incl: survival
Salinas-Escudero, 2012	Mexico	UMIC	Cost-effectiveness economic evaluation via clinical record review				
Ozkan, 2018	Turkey	UMIC	Guideline				Revision of national guideline for RDS & SRT
Salinas-	Mexico	UMIC	Cost-effectiveness				

**Author**	**Country**	**WBI group**	**Methodology**	**Population**	**Intervention**	**Control**	**Primary Outcome(s)**
Escudero, 2012			study				

Supplementary Table 3: Included studies (Systematic reviews)

Author, year	Research question	Countries of included studies	WBI group	Databases	Study type, search limits	Overall sample	PICO
Wu, 2021	Efficacy & safety of LISA in neonatal respiratory distress syndrome (NRDS)	Germany (2), Iran (4), Turkey (1), China (3), Pakistan (1), India (2)	HIC (2), UMIC (4), LMIC (7)	PubMed, EMBASE, Cochrane Library and Web of Science	Published clinical RCTs, in English<br><br>Inception - 11/2020	Total included studies, n = 13<br>- total population, n = 1931<br>- intervention group, n = 964<br>- control group, n = 967	P - GA <37w, NRDS<br>I - LISA via thin catheter<br>C -INSURE<br>O - ≥1 of: mortality rate, requirement for IMV, BPD, ROP, IVH, NEC & other neonatal morbidity
Zhong, 2019	Efficacy & safety of early airway administration of corticosteroids (ICS) & SRT for preventing BPD in NRDS	China (7), Iran (1), USA (1)	UMIC (7), LMIC (1), HIC (1)	PubMed, Web of Science, EMBASE, Cochrane Library, Clinicaltrials.gov, Controlled-trials.com, Google Scholar, VIP, Wanfang and proceedings of the Pediatric Academic Society meetings from database	Clinical RCTs<br><br>Inception - 08/2018	Total included studies, n = 15<br>- total population, n = 792<br>- intervention group, n = 414<br>- control group, n = 378	P - GA <36w, NRDS<br>I - ICS + SRT, within 1 day after birth<br>C - placebo + SRT, within 1 day after birth<br>O - >1:<br>Primary: BPD incidence
Cao, 2020	Potential effect of LISA procedure on RDS in preterm neonates	Iran (2), Turkey (1), China (4), Germany (3)	LMIC (2), UMIC (5), HIC (3)	PubMed, Embase, Cochrane<br>Wanfang, VIP, and Cnki databases	Human clinical studies<br>English, Mandarin &	Total included studies, n = 10<br>- total population, n = 3341	P - not specified<br>I - LISA<br>C - not specified

Author, year	Research question	Countries of included studies	WBI group	Databases	Study type, search limits	Overall sample	PICO
					other languages<br>Inception - 12/2018	- intervention group, n = 1666<br>- control group, n = 1675	O - MV, FiO2, BPD, hospital stay, complications of RDS, death
Sankar, 2016	(1) Efficacy & safety of SRT, and (2) feasibility and cost-effectiveness of introducing and implementing SRT in LMIC settins	Mexico (1), Turkey (3), South Africa (4), Five Latin American countries (1), Malaysia (5), Brazil (5), Chile (2), Curacao (1), Argentina (1), India (3), China (6), Four South American countries (Argentina, Chile, Peru and Uruguay) (1), South American countries (Argentina and Chile) (1), Iran (1), Thailand (1)	UMIC & LMIC	Objective 1: MEDLINE, Cochrane CENTRAL, EMBASE, CINAHL<br><br>Objective 2: PubMed, Cochrane CENTRAL, WHOLIS	Objective 1: Observational & experimental studies comparing effects of SRT with no or placebo therapy in preterm neonates w RDS<br><br>Objective 2: All studies reporting use of SRT in preterm neonates with or at-risk of RDS<br><br>No language restrictions<br><br>1998 - 07/13	Total included studies, n = 38<br>- 2 RCTs<br>- 12 before-and-after<br>- 8 concurrent control & case-control<br>- 8 case-series	P - preterm neonates with or at risk of RDS & req SRT<br>I - single or multiple doses SRT by intra-tracheal route<br>C - no or placebo treatment<br>O - neonatal mortality, in-hospital mortality, BPD, air leaks, incidence/prevalence of pulmonary haemorrhage, incidence/prevalence of complications such as apnea, hypoxia/arrest during or immediately after SRT, proportion of neonates who received the entire dose of surfactant

**Author, year**	**Research question**	**Countries of included studies**	**WBI group**	**Databases**	**Study type, search limits**	**Overall sample**	**PICO**
					(updated 12/14)<br>(EMBASE & CINAHL 2007-2013 only)		successfully, proportion of neonates wh needed referral to higher centers immediately after SRT, cost-effectiveness
Rong, 2020	Effects & safety of nebulized vs invasively delivered SRT in treatment of NRDS	China (2)	UMIC (2)	PubMed, Embase, and the Cochrane Library, China National Knowledge Infrastructure (CNKI) and Wanfang Database, China Biomedical Literature Database (CBM)	Clinical RCTs<br>No language restrictions<br>Inception - May 2020	Total included studies, n = 2<br>- total population, n = 95<br>- intervention group, n = 48<br>- control group, n = 47	P - neonates w RDS requiring SRT<br>I - nebulized SRT<br>C - invasively delivered SRT<br>O - SpO2 level 1 h after treatment, gas analysis results (A/APaO2 level 1 h after treatment), length of MV
Panza, 2020	Review data on current best surfactant delivery methods	India, Pakistan, German Neonatal Network, Australia, China, Turkey, Austria	HICs + UMICs + LMICs	PubMed, Embase, Cochrane Library and Web of Science. Additional studies identified from trial registries, conference proceedings & reference lists	RCTs, observational studies & feasibility studies<br>Published in English Language	Total included studies, n = 15<br>- RCTs, n =6<br>- observational, n = 7<br>- feasibility studies, n = 2<br>- overall dataset (SRT given by LISA), n =	P - neonates w RDS requiring SRT<br>I - SRT via thin catheter (LISA)<br>C - SRT via ETT (INSURE)<br>O - need for MV, BPD

Author, year	Research question	Countries of included studies	WBI group	Databases	Study type, search limits	Overall sample	PICO
					Inception - 11/19	4926	
Zhang, 2015	Evaluate effects of 6 exogenous surfactants on mortality rate in NRDS	USA (9), Turkey (1), Iran (1), Germany (2), Greece (2), Finland (1), Sultanate of Oman (1)	HICs + UMICs + LMICs	PubMed, Ovid, EBSCO, Springerlink, Wiley, Web of Science, Cochrane Library, China National Knowledge Infrastructure, Wanfang and VIP databases	RCTs<br><br>No language restrictions<br><br>1995 - 2013 (updated 10/14)	Total studies included, n = 52; qualitative, n = 35, quantitative, n = 17<br><br>- overall sample size, n = 57,223 infants w NRDS	P - infants 23-36w w NRDS confirmed by clinical diagnosis<br><br>I - treatment w Survanta or Alveofact or Infasurf or Curosurf or Surfaxin or Exosurf for NRDS<br><br>C - not specified<br><br>O - mortality rate
Yeung, 2023	Efficacy & safety of SRT via thin catheter (STC) compared to intubation for surfactant or nCPAP	Germany (2), Turkey (1), Iran (6), China (8), Quebec (1), India (4), Pakistan (2), Egypt (1)	HICs + UMICs + LMICs	Medline, Excerpta Medica database (Embase), Cumulative Index of Nursing and Allied Health Literature (CINAHL), Cochrane Central Register of Controlled Trials (CENTRAL) databases and the China National Knowledge Infrastructure	RCTs<br><br>No language restrictions<br><br>Inception - 12/22	Total studies included, n = 26	P - Preterm (<37+0w) infants with or at-risk of RDS and req SRT<br><br>I - STC that involved passing a thin catheter with direct or video laryngoscopy beyond the cords for SRT while on NIV<br><br>C - Infants treated with INSURE, or intubation for surfactant and MV w

Author, year	Research question	Countries of included studies	WBI group	Databases	Study type, search limits	Overall sample	PICO
				Database (CNKI); in addition, the first 400 hits in Google Scholar were searched for articles that may not have been indexed in the standard medical databases			delayed extubation, or nCPAP alone<br>O - BPD at 36w gestation in survivors
Tang 2021	Effectiveness and safety of early combined utilization of budesonide and surfactant by airway to prevent BPD in preterm infants w RDS	China (11), Taiwan (1), US & Taiwan (1), Iran (2), Korea (1)	HIC (3) + UMIC (11) + LMIC (2)	PubMed, Web of Science, EMBASE, Cochrane Library, Wanfang, CQVIP and China National Knowledge Infrastructure (CNKI)	RCTs<br>No language restrictions<br>Inception - 09/21	Total studies included, n = 17<br>-overall sample, n = 1735<br>- BUD group, n = 858<br>- control group, n = 877	P - GA<33w or BW <1500g + RDS, received intervention <8d of life<br>I - budesonide + surfactant administered<br>C - surfactant alone administered<br>O - BPD incidence
De Luca, 2021	Limitations in terms of "lack of solid pathobiological and physiological background supporting LISA" via metanalysis and synthesis of grouped studies	Germany (2), Turkey (1), Iran (2), China (4), Pakistan (1), India (2)	HIC (2) + UMIC (4) + LMIC (5)	Method not elucidated; however, meta-analyses done	2011 - 2021	Total studies included, n = 12	P - preterm neonates w RDS (without other co-existing disorders)<br>I - LISA<br>C - INSURE<br>O - meta-analysis done for mortality,

Author, year	Research question	Countries of included studies	WBI group	Databases	Study type, search limits	Overall sample	PICO
							BPD incidence, need for MV in first 72 h
Abdel-Latif, 2021	Primary Objective: Compare SRT via thin catheter (S-TC) with INSURE or continuation NIV w no SRT, or intubation	China (3), Iran (5), Netherlands (1), India (2), Germany (2), Pakistan (1), Turkey (1), Canada (1)	HICs (5) + UMICs (4) + LMICs (8)	Standard search strategy of Cochrane Neonatal CENTRAL, in the Cochrane Library; Ovid MEDLINE (R) and Epub Ahead of Print, In-Process & Other Non-Indexed Citations, Daily and Versions (R); and CINAHL; clinical trials databases (ISRCTN registry, ICTRP); reference lists; expert informants; previous reviews; unpublished trials if final trial data available	RCTs, parallel intervention trials, randomised or quasi-randomised studies<br><br>1990 - 09/20	Total included publications, n = 18<br>- overall sample, n = 2164<br><br>Studies, (n)<br>- S-TC vs S-ETT (12)<br>- S-TC vs S-ETT w delayed extubation (2)<br>- S-TC vs continuation NIV (1)<br>- MIST w sedation vs MIST without sedation (1)	P - GA <37w with or at-risk RDS<br>I: S-TC<br>C: S-ETT, or NIV ± SRT or MV, or comparison of strategies of S-TC<br>O - death or BPD at 36 weeks' PMA, need for MV within 72 h of birth, air leak requiring draiange, severe IVH (gr III or IV), death during first hospitalization, BPD among survivors, death or survival w neurosensory disability

Supplementary Table 4. Characteristics of surfactant used in included studies

Surfactant type & Dose	RCTs<br>n/38φ (%)	Observational<br>n/46φ* (%)	Total<br>n/83 (%)
Poractant alfa 100mg/kg	5 (13.2)	1 (2.2)	6 (7.2)
Poractant alfa 200mg/kg	16 (42.1)	7 (15.6)	23 (27.7)
Poractant alfa 100-200mg/kg	0	2 (4.4)	2 (2.4)
Poractant alfa 50-240mg/kg	0	1 (2.2)	1 (1.2)
Beractant 100mg/kg	8 (21.1)	6 (13.3)	14 (16.9)
Bovine (NOS) 100mg/kg	0	1 (2.2)	1 (1.2)
Neosurf 135mg/kg	1 (2.6)	0	1 (1.2)
Calsurf 70-100mg/kg	3 (7.9)	0	3 (3.6)
Beractant 100mg/kg *or* Poractant alfa 100mg/kg	1 (2.6)	1 (2.2)	2 (2.4)
Beractant 100mg/kg *or* Poractant alfa 200mg/kg	0	1 (2.2)	1 (1.2)
**Only type specified**			
Poractant alfa	1(2.6)	0	1(1.2)
**Only dose specified**			
100mg/kg	1(2.6)	2(4.4)	3(3.6)
200mg/kg	0	1(2.2)	1(1.2)
1.25-2.5mL/kg	0	1(2.2)	1(1.2)

3mL/kg	0	1(2.2)	1(1.2)
**Neither type nor dose specified**	2(5.3)	20(44.4)	22(26.5)
**Total**	38(100)	46(100)	

φStudies that directly compared surfactant types excluded from above (analysed separately)

*Cost-effectiveness & diagnostic accuracy studies, and guidelines excluded

Supplementary Table 5. Studies comparing types of surfactant

Author(s) (year)	Comparison surfactant types	RCTs (n)	Observational studies (n)
Yilmaz (2021)	Beractant 100mg/kg<br>Poractant alfa 100mg/kg<br>Infasurf (Calfactant) 100mg/kg	1	
Jain (2019)	GLSE dose NS<br>Beractant dose NS	1	
Terek (2015); Gharehbaghi (2010); Karadag (2016); Bozkaya (2021); Dizdar (2012); Şimşek (2020)	Beractant 100mg/kg<br>Poractant alfa 200mg/kg	5	1
Mirzarahimi (2018); Najafian (2016); Bozdağ (2015)	Beractant 100mg/kg<br>Poractant alfa 100mg/kg	3	
Mussavi (2016)	Alveofact 100mg/kg<br>Poractant alfa 200mg/kg<br>Beractant 100mg/kg	1	

	Butantan 100mg/kg		
	Beractant 100mg/kg		
Rebello (2014)	Poractant alfa 100mg/kg	1	
	BLES 100mg/kg		
Macooie (2018)	Beractant 100mg/kg	1	
	BLES 135mg/kg		
Sarokolai (2018)	Poractant alfa 200mg/kg	1	
	Beractant 100mg/kg		
Patel (2022)	Synthetic 67.5mg/kg (5mL/kg)		1

Appendix 2: Supplementary Figures

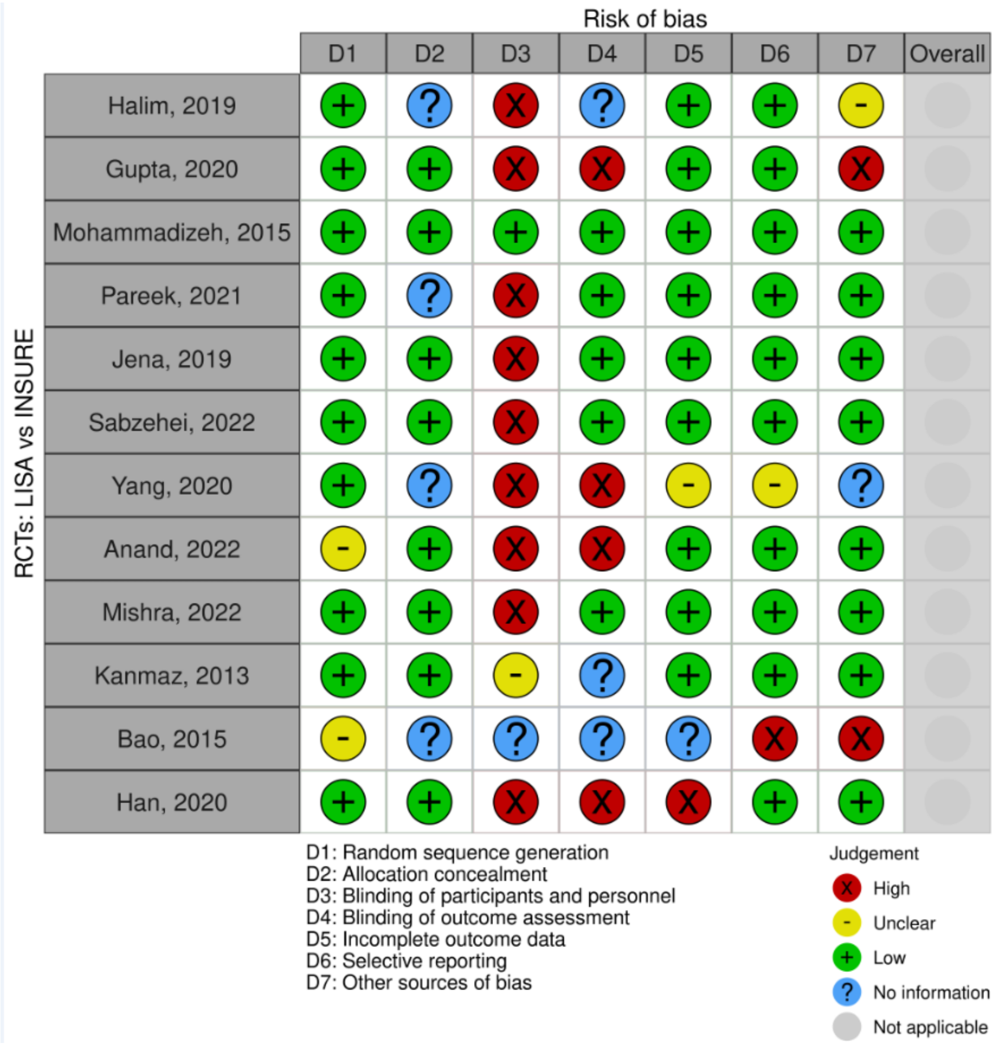

Supplementary figure 1. Critical appraisal infographic for pooled studies regarding method of SRT delivery

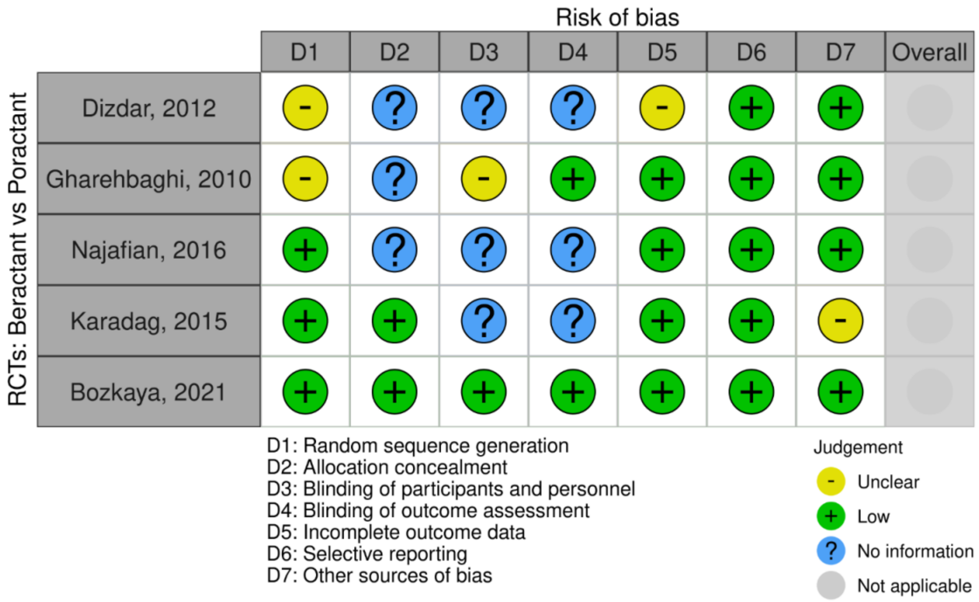

Supplementary Figure 2: Critical appraisal infographic for pooled studies regarding surfactant type

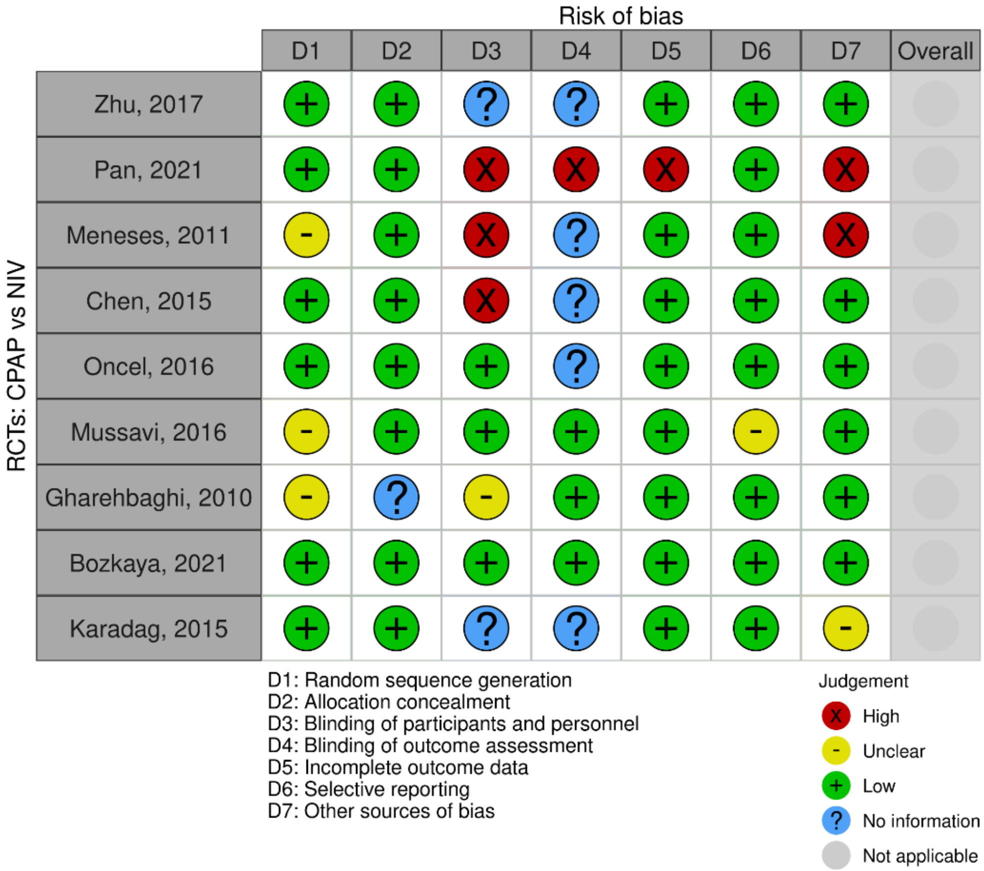

Supplementary Figure 3. Critical appraisal infographic for pooled studies regarding CPAP vs NIV

Appendix 3:

HREC Ethics Waiver Letter

UNIVERSITY OF CAPE TOWN
Faculty of Health Sciences
Human Research Ethics Committee

Room G50-46 Old Main Building
Groote Schuur Hospital
Observatory 7925
Telephone [021] 406 6492
Email: hrec-enquiries@uct.ac.za
Website: www.health.uct.ac.za/fhs/research/humanethics/forms

17 May 2021

HREC/REF 301/2021

Dr L Tooke
Division of Neonatology
H-Floor-, OMB
Email: lloyd.tooke@uct.ac.za
Student: Caris.ronan@outlook.com

Dear Dr Tooke

PROJECT TITLE: USE OF SURFACTANT REPLACEMENT THERAPY AND ASSOCIATED STRATEGIES IN PRETERM OR LOW BIRTH WEIGHT NEONATES IN LOW- AND MIDDLE-INCOME COUNTRIES – A SYSTEMATIC DESCRIPTIVE REVIEW-MASTERS CANDIDATE-DR CARIS PRICE

Thank you for submitting your request to the Faculty of Health Sciences Human Research Ethics Committee.

The HREC note that the proposed study is a systematic review.

As the systematic review involves published literature available through publicly accessible electronic databases, research ethics review and approval is not required.

This is in accordance with Section 1.1.8 of the Department of Health's Ethics in Health Research: Principles, Processes and Structures (South African Department of Health, 2015), which states: "*Research that relies exclusively on publicly available information or accessible through legislation or regulation usually need not undergo formal ethics review. This does not mean that ethical considerations are irrelevant to the research.*"

The HREC recommend that researchers refer to the PRISMA website, for the PRISMA statement and checklist, to facilitate the reporting of systematic reviews and meta-analyses. For more information, please refer to http://www.prisma-statement.org/.

Further, fundamental ethical principles for health-related research should be considered in the objectives and methods of the systematic review. See, for example, the Declaration of Helsinki (Fortaleza, Brazil, 2013) and the Department of Health's Ethics in Health Research: Principles, Processes and Structures (South African Department of Health, 2015).

The HREC acknowledge that the Master's candidate, Dr Caris Price, is also involved in this project.

Yours sincerely

PROFESSOR M BLOCKMAN
CHAIRPERSON, FACULTY OF HEALTH SCIENCES HUMAN RESEARCH ETHICS COMMITTEE
